# Chocolate intake and risk of type 2 diabetes: prospective cohort studies

**DOI:** 10.1136/bmj-2023-078386

**Published:** 2024-12-04

**Authors:** Binkai Liu, Geng Zong, Lu Zhu, Yang Hu, JoAnn E Manson, Molin Wang, Eric B Rimm, Frank B Hu, Qi Sun

**Affiliations:** 1 Department of Nutrition, Harvard T.H. Chan School of Public Health, Boston, MA, USA; 2 Key Laboratory of Nutrition, Metabolism and Food Safety, Shanghai Institute of Nutrition and Health, University of Chinese Academy of Sciences, Chinese Academy of Sciences, Shanghai, China; 3 Department of Endocrinology and Metabolism, Shanghai Jiao Tong University Affiliated Sixth People’s Hospital, Shanghai, China; 4 Department of Epidemiology, Harvard T.H. Chan School of Public Health, Boston, MA, USA; 5 Channing Division of Network Medicine, Department of Medicine, Brigham and Women’s Hospital and Harvard Medical School, Boston, MA, USA; 6 Division of Preventive Medicine, Department of Medicine, Brigham and Women’s Hospital and Harvard Medical School, Boston, MA, USA; 7 Department of Biostatistics, Harvard T.H. Chan School of Public Health, Boston, MA, USA

## Abstract

**Objective:**

To prospectively investigate the associations between dark, milk, and total chocolate consumption and risk of type 2 diabetes (T2D) in three US cohorts.

**Design:**

Prospective cohort studies.

**Setting:**

Nurses’ Health Study (NHS; 1986-2018), Nurses’ Health Study II (NHSII; 1991-2021), and Health Professionals Follow-Up Study (HPFS; 1986-2020).

**Participants:**

At study baseline for total chocolate analyses (1986 for NHS and HPFS; 1991 for NHSII), 192 208 participants without T2D, cardiovascular disease, or cancer were included. 111 654 participants were included in the analysis for risk of T2D by intake of chocolate subtypes, assessed from 2006 in NHS and HPFS and from 2007 in NHSII.

**Main outcome measure:**

Self-reported incident T2D, with patients identified by follow-up questionnaires and confirmed through a validated supplementary questionnaire. Cox proportional hazards regression was used to estimate hazard ratios and 95% confidence intervals (CIs) for T2D according to chocolate consumption.

**Results:**

In the primary analyses for total chocolate, 18 862 people with incident T2D were identified during 4 829 175 person years of follow-up. After adjusting for personal, lifestyle, and dietary risk factors, participants consuming ≥5 servings/week of any chocolate showed a significant 10% (95% CI 2% to 17%; P trend=0.07) lower rate of T2D compared with those who never or rarely consumed chocolate. In analyses by chocolate subtypes, 4771 people with incident T2D were identified. Participants who consumed ≥5 servings/week of dark chocolate showed a significant 21% (5% to 34%; P trend=0.006) lower risk of T2D. No significant associations were found for milk chocolate intake. Spline regression showed a linear dose-response association between dark chocolate intake and risk of T2D (P for linearity=0.003), with a significant risk reduction of 3% (1% to 5%) observed for each serving/week of dark chocolate consumption. Intake of milk, but not dark, chocolate was positively associated with weight gain.

**Conclusions:**

Increased consumption of dark, but not milk, chocolate was associated with lower risk of T2D. Increased consumption of milk, but not dark, chocolate was associated with long term weight gain. Further randomized controlled trials are needed to replicate these findings and further explore the mechanisms.

## Introduction

The global prevalence of type 2 diabetes (T2D) has increased noticeably over the past few decades, with an estimated 463 million people affected worldwide in 2019 and projected to increase to 700 million by 2045.^1^ T2D is a multifactorial disease characterized by insulin resistance and impaired insulin secretion, which can lead to numerous severe complications such as cardiovascular disease, renal failure, and loss of vision.^2^ A growing body of research has highlighted the importance of lifestyle factors, including healthy diets, in the prevention and management of T2D.^3^ ^4^

Higher consumption of total dietary flavonoids, as well as specific flavonoid subclasses, has been associated with a decreased risk of T2D.^5^ ^6^ In randomized controlled trials, these flavonoids exerted antioxidant, anti-inflammatory, and vasodilatory effects that might confer cardiometabolic benefits and reduce the risk of T2D,^7^ ^8^ ^9^ although data were not consistent.^10^ Chocolate, derived from the beans of the cacao tree (*Theobroma cacao*), is among foods with the highest flavanol content and is a popular snack globally.^11^ ^12^ ^13^ However, the association between chocolate consumption and risk of T2D remains controversial owning to inconsistent findings in observational studies.^14^ ^15^ Furthermore, most previous studies have primarily focused on total chocolate intake, without considering the potential differences in health effects between chocolate subtypes (ie, dark, milk, and white chocolate).^16^ ^17^ These subtypes differ in cocoa content and proportions of other ingredients such as sugar and milk, which may influence the association with risk of T2D.^18^

Using data from three prospective cohort studies that repeatedly assess participants’ diet during longitudinal follow-up, we investigated the association between subtypes of chocolate intake and risk of T2D, as well as change in bodyweight, which is a strong predictor for risk of T2D.^19^

## Methods

### Study population

This study used data from three large prospective cohorts: the Nurses’ Health Study (NHS), initiated in 1976 and comprising 121 700 female registered nurses^20^; the Nurses’ Health Study II (NHSII), launched in 1989 and comprising 116 340 female nurses^21^; and the Health Professionals Follow-Up Study (HPFS), initiated in 1986 and comprising 51 529 male health professionals.^22^

For analyses on total chocolate consumption, the study baselines (baseline 1) were 1986 for NHS and HPFS and 1991 for NHSII, when diet was first comprehensively assessed using a validated semiquantitative food frequency questionnaire.^23^ ^24^ ^25^ For analyses on chocolate subtypes, the study baselines (baseline 2) were 2006 for NHS and HPFS and 2007 for NHSII, coinciding with the availability of data on chocolate subtypes in the food frequency questionnaire.

In the primary analyses, we excluded participants who had prevalent diabetes, cardiovascular disease (myocardial infarction, coronary artery bypass surgery, stroke), or cancer (except non-melanoma skin cancer) at baseline; had missing information on baseline age and chocolate intake, or an unusual total energy intake (ie, <600 or >3500 kcal/d for women and <800 or >4200 kcal/d for men); only completed the baseline food frequency questionnaire; or had an undetermined diagnosis date for T2D.

In a secondary analysis of change in bodyweight by levels of chocolate intake, we excluded participants with self-reported diabetes, cardiovascular disease, or cancer at baseline. Additional baseline exclusions were extreme total energy intake and missing data on chocolate intake or bodyweight. During follow-up, we censored individuals who reported cardiovascular disease, cancer, or a diagnosis of diabetes. We also censored individuals with missing data on chocolate intake or bodyweight during follow-up. The flowchart in supplementary figure S1 shows the selection process for participants.

The study protocol was approved by the Human Research Committee of Brigham and Women’s Hospital and the Harvard T.H. Chan School of Public Health. Participants provided informed consent by completing and returning study questionnaires.

### Assessment of diet

Diet was assessed every four years using a semiquantitative food frequency questionnaire.^23^ Participants reported their average frequency of consumption for a standard food portion size (eg, one chocolate bar/pack or 1 oz) in the past year, choosing from nine levels ranging from “never, or less than once per month” to “≥6 per day.” Questions about chocolate consumption were included from 1980 in NHS (study baseline was set at 1986 for total chocolate when the comprehensive food frequency questionnaire was first administered in NHS), 1991 in NHSII, and 1986 in HPFS. Questions about specific subtypes of chocolate consumption, specifically “How often do you consume milk chocolate (bar or pack),” and “How often do you consume dark chocolate,” were added to the food frequency questionnaire from 2006 for NHS and HPFS and from 2007 for NHSII, which were study baselines for the analyses by chocolate subtypes. Information on consumption of chocolate subtypes was collected in 2006 and 2010 for NHS; 2007, 2001, and 2015 for NHSII; and 2006, 2010, 2014, and 2018 for HPFS. Nutrient intakes were measured based on the US Department of Agriculture food composition database. We calculated the average daily intake of nutrients by multiplying the frequency of consumption of each food item that contains the nutrient by the nutrient content, and then summing across all food items. The intake level of flavan-3-ols was calculated by summing the individual components (catechin, epicatechin, gallocatechin, epigallocatechin, epicatechin 3-gallate, and epigallocatechin 3-gallate). The intake level of total flavonoids was further calculated by summing intakes of flavonols, flavones, flavanones, flavan-3-ols, total theaflavins, and polymers proanthocyanidins. Total energy intake was derived from the food frequency questionnaires using the same algorithm.

The validity and reproducibility of the food frequency questionnaires have been reported in detail previously.^23^ ^24^ ^25^ In validation studies among subgroups of participants in NHS and HPFS, the correlation coefficient between chocolate intake assessed by diet records over seven days and food frequency questionnaire was, respectively, 0.53 and 0.65 for dark chocolate and 0.42 and 0.47 for milk chocolate.^26^ Additionally, the food frequency questionnaire shows reasonable consistency of food intake measurements over time, with mean intraclass correlation coefficients of 0.71 (dark: 0.71; milk: 0.59) in NHS and 0.72 (dark: 0.66; milk: 0.60) in HPFS.^26^

### Assessment of covariates

In all three cohorts, information on participants’ personal and lifestyle factors, as well as diseases or medical conditions, was collected biennially through questionnaires. Variables assessed included race/ethnicity, bodyweight, waist circumference, smoking status, alcohol consumption, multivitamin use, menopausal status and postmenopausal hormone use (women only), oral contraceptive use (NHSII only), hypertension, hypercholesterolemia, and family history of diabetes. Specialty of health professionals was assessed in HPFS, and academic or professional degrees earned were assessed in NHS. Physical activity was measured using a validated questionnaire,^27^ ^28^ ^29^ which derived metabolic equivalent tasks in hours per week based on time spent on 10 recreational activities. Height was measured in the baseline questionnaire of these three cohorts, and time varying body mass index (BMI) was calculated as biennially updated bodyweight in kilograms divided by the square of height in meters. The Alternate Healthy Eating Index-2010 (AHEI) was calculated based on participants’ responses in the food frequency questionnaire to reflect overall diet quality, which emphasizes foods and nutrients, such as whole grains, nuts, red and processed meats, sugar sweetened beverages, and alcohol, that predict the risk of chronic disease.^30^ For NHS and NHSII, we further calculated z scores of standardized neighborhood socioeconomic status using census tract variables from the Neighborhood Change Database. More details on computation are described elsewhere.^31^

### Assessment of T2D

T2D was self-reported in the biennial follow-up questionnaires, and the diagnosis was further confirmed by study doctors through a supplementary questionnaire, which collected data on factors such as glucose concentration, glycated hemoglobin (HbA_1c_) concentration (after 2010), symptoms and treatments for T2D. People with confirmed T2D, which constituted the outcome of this analysis, were defined according to the National Diabetes Data Group criteria before 1998,^32^ where at least one of the following items was reported: raised fasting plasma glucose level (≥7.8 mmol/L (140 mg/dL)) or plasma glucose (≥11.1 mmol/L (200 mg/dL)), and at least one T2D symptom (excessive thirst, polyuria, weight loss, hunger); no T2D symptom, but had at least two raised plasma glucose measurements on different occasions; or treatment with insulin or other hypoglycemic drug (insulin or oral hypoglycemic agent). For people with T2D diagnosed after 1998, the threshold for raised fasting plasma glucose level was lowered to 7.0 mmol/L (126 mg/dL) according to the criteria of the American Diabetes Association.^33^ For people with a diagnosis after January 2010, we further added HbA_1c_ ≥6.5% to the diagnostic criteria.^34^ The validity of the supplementary questionnaire was established in two previous studies in NHS and HPFS through blinded medical record reviews, with T2D diagnosis confirmed in 98% and 97% of people, respectively.^35^ ^36^

### Ascertainment of weight change

Bodyweight was self-reported at baseline and in the biennial follow-up questionnaires, and it has been previously validated in these cohorts, with correlation coefficients of 0.97 in NHS and HPFS and 0.84 in NHSII.^37^ ^38^ We calculated the outcome of interest, weight change every four years, by subtracting bodyweight at the start of each four year interval from bodyweight at the end of the four year interval.

### Statistical analysis

We present participants’ characteristics at baseline 1 (1986 for NHS and HPFS, 1991 for NHSII) and baseline 2 (2006 for NHS and HPFS, 2007 for NHSII), stratified by consumption levels of total, dark, and milk chocolate and further categorized by study cohort. This breakdown allowed for a more nuanced understanding of the characteristics of participants, as individuals consuming similar levels of dark or milk chocolate might have distinct baseline characteristics across cohorts. To reflect the long term usual intake of total chocolate at baseline 2, we calculated cumulatively averaged total chocolate intake at or before baseline 2 (1980-2006 for NHS, 1991-2007 for NHSII, and 1986-2006 for HPFS). Person years were calculated as the duration between the return date of the baseline food frequency questionnaire to the date of T2D diagnosis, date of death, date of the last return of a valid follow-up questionnaire, or end of follow-up (2018 for NHS, 2021 for NHSII, and 2020 for HPFS), whichever came first. To reduce potential reverse causality that participants changed their diet after diagnosis of certain diseases, we stopped diet updates once participants reported myocardial infarction, stroke, coronary artery bypass graft, or cancer during follow-up. The rates for missing covariates were low in the cohorts. Any missing values for physical activity, alcohol intake, total energy intake, food and nutrients intake, BMI, AHEI, or z scores for socioeconomic status during follow-up were first replaced by valid values in the previous questionnaire cycle. For the remaining missing data of individual food items (<0.5%), we replaced the missing values with the median intake in the cohort. For missing values of smoking status, oral contraceptive use, menopausal status, and postmenopausal hormone use, we used the missing covariate indicator method.

In primary analyses, we used Cox proportional hazards models to estimate the hazard ratios and corresponding 95% confidence intervals (CIs) for the association between consumption levels of total, dark, and milk chocolate and risk of T2D.^39^ To maximize statistical power and reduce within person variations, we cumulatively averaged total chocolate intake from baseline 1 and dark and milk chocolate intake from baseline 2. We categorized regular chocolate consumption into four groups of levels, including never or <1 serving/month (reference level), 1 serving/month to <1 serving/week, 1-4 servings/week, and ≥5 servings/week. The Cox models were stratified by age and calendar time. Fully adjusted models accounted for age, calendar year, ethnicity (white, African American, Asian, and other ethnicities), smoking status (never, former, current (cigarettes/day: 1-14, 15-24, or ≥25), or missing), alcohol intake (g/day: 0, 0.1-4.9, 5.0-14.9, and ≥15.0 in women, 0, 0.1-4.9, 5.0-14.9, 15.0-29.9, and ≥30.0 in men, or missing), family history of diabetes (yes/no), menopausal status and postmenopausal hormone use (premenopausal, postmenopausal (never, former, or current hormone use), or missing, for women), use of oral contraceptives (yes, no, NHSII only), physical activity (metabolic equivalent task hours (MET-h)/week: <3, 3.0-8.9, 9.0-17.9, 18.0-26.9, ≥27.0, or missing), baseline BMI (<21.0, 21.0-22.9, 23.0-24.9, 25.0-26.9, 27.0-29.9, 30.0-32.9, 33.0-34.9, ≥35.0, or missing), multivitamin use (yes/no), baseline hypertension, baseline hypercholesterolemia, total energy intake (continuous, kcal/day), and AHEI (fifths). For analyses of chocolate subtypes, we further adjusted total chocolate intake before baseline in the models (as cumulative averages for 1980-2002 (NHS), 1991-2003 (NHSII), 1986-2002 (HPFS)). Analyses were run separately in each cohort first, and then data were pooled to calculate overall study estimates, with cohort origin further adjusted in the model.

Sensitivity analyses were conducted using the pooled dataset: using baseline chocolate intake level instead of time varying intake; using most recently updated data on chocolate intake instead of cumulatively averaged intake; adjusting for baseline BMI as a continuous instead of categorical variable; adjusting for time varying BMI instead of baseline BMI to explore the mediation effect of BMI; adjusting for time varying waist circumference instead of baseline BMI; adjusting for consumption of specific food and beverages high in flavan-3-ols (eg, tea, red wine, apples, pears, blueberries, bananas, and peppers) instead of AHEI; adjusting for consumption of red and processed meat, fruit and vegetables, sugar sweetened beverages, and whole grains instead of AHEI; further adjusting for each individual intake of flavonoids; further adjusting for saturated fat intake; and further adjusting for added sugar intake. In the pooled dataset, we examined potential effect modification by age (<70 years, ≥70 years), sex (male, female), baseline BMI (<30, ≥30), physical activity (<median, ≥median), AHEI (<median, ≥median), and family history of diabetes (yes, no). Stratified analyses were conducted for these binary modifiers, and all models were multivariable adjusted. To address any residual confounding by BMI, we further adjusted baseline BMI as a continuous variable in the BMI stratified models. Interaction terms were created between the binary effect modifiers and the categorical chocolate consumption levels, and P values for interaction were examined using the likelihood ratio test.

Moreover, we explored the potential dose-response association between intake of total, dark, and milk chocolate and risk of T2D using restricted cubic spline analysis (SAS Macro %LGTPHCURV9)^40^ with three knots, adjusting for the previously mentioned covariates. To reduce the effect of outliers, we combined and truncated data from the three cohorts at the first and 99th centiles. We performed a sensitivity analysis of spline regression not excluding outliers. Additionally, linear regressions were run between total, dark, and milk chocolate intake with selected nutrients, food, and drink at baseline, such as saturated fat, added sugar, flavan-3-ols, and sweets and desserts.

To further determine the association of chocolate intake with weight change, we conducted secondary analyses using multivariable generalized linear regression models with independent correlation matrix and robust variance to examine the associations of four year changes in chocolate intake with concomitant four year changes in bodyweight. For total chocolate analyses, eight four year cycles were available for HPFS (1986-2018) and six four year cycles for both NHS (1986-2010) and NHSII (1991-2015). For analyses of chocolate subtypes, three four year cycles were available for HPFS (2006-18), one four year cycle for NHS (2006-10), and two four year cycles for NHSII (2007-15). For each four year interval, we examined the associations between chocolate intake (increased or decreased versus no change) and weight change. We combined data from three cohorts for this analysis. In the multivariable adjusted model, we adjusted for age, ethnicity (white, African American, Asian, other), family history of diabetes (yes/no), baseline hypertension (yes/no), baseline hypercholesterolemia (yes/no), baseline total energy intake (continuous, kcal/day), postmenopausal hormone use (women only; premenopausal, never, former, current, or missing; time varying), and oral contraceptive use (NHSII only; yes, no; time varying), baseline and change in physical activity (MET-h/week), change in smoking status (stayed never smoker, stayed former smoker, stayed current smoker, changed from former to current smoker, changed from never to current smoker, and changed from current to former smoker), change in alcohol consumption (continuous, g/day), change in AHEI (fifths), and study origin (NHS, NHSII, HPFS). We also conducted stratified weight change analyses by baseline BMI (<25.0, 25.0-29.9, or ≥30.0) to explore any potential effect modifications. We adjusted for the same covariates as in the main analysis, and we further adjusted for baseline BMI in the continuous form. P values for interactions were calculated by general score tests requested by the type 3 command in the model statement (equivalent to the likelihood ratio test).

Analyses were conducted using SAS for Unix (version 9.4, SAS Institute) and RStudio (version 4.2.3, RStudio). We considered two sided P values <0.05 to be statistically significant.

### Patient and public involvement

We did not have the infrastructure, resources, funding, or time to involve the public in study design, interpretation of results, or publication.

## Results

### Baseline characteristics

Supplementary figure S1 shows the selection of participants at specific baselines for primary analysis. Table 1, table 2, and supplementary table S1 present age standardized characteristics of participants at baselines 1 and 2, stratified by cohort, chocolate consumption levels, and types of chocolate (total, dark, and milk, respectively). A total of 192 208 participants (63 798 women in NHS, 88 383 women in NHSII, and 40 027 men in HPFS) were included in the analysis of total chocolate intake and 111 654 (39 400 women in NHS, 58 187 women in NHSII, and 14 067 men in HPFS) in the analyses of chocolate subtypes. The mean ages at baseline 1 were 52.3 years in NHS, 36.1 years in NHSII, and 53.1 years in HPFS. The mean ages at baseline 2 were 70.4 years in NHS, 52.3 years in NHSII, and 68.3 years in HPFS. Most participants were of non-Hispanic white ethnicity. Across all three cohorts, participants with higher levels of chocolate intake also had higher energy intakes, saturated fat, and added sugar. Higher consumption of dark chocolate was associated with higher AHEI and consumption of fruit and vegetables, epicatechin, and total flavonoids. Associations between milk chocolate consumption and these dietary variables were, however, inverse. The distribution of participants’ characteristics across total chocolate intake groups were similar to those for milk chocolate groups.

**Table 1:** Age standardized baseline characteristics of participants according to dark chocolate* intake at baseline. Values are mean (SD) unless stated otherwise

	Frequency of dark chocolate intake													
	NHS					NHSII					HPFS			
	0 or <1 serving/month	1 serving/month to <1 serving/week	1-4 servings/week	≥5 servings/week		0 or <1 serving/month	1 serving/month to <1 serving/week	1-4 servings/week	≥5 servings/week		0 or <1 serving/month	1 serving/month to <1 serving/week	1-4 servings/week	≥5 servings/week
No of participants	25 476	7591	5363	970		27 471	16 098	11 944	2674		7395	3582	2555	535
Servings/week:														
Dark chocolate	0 (0)	0.5 (0)	1.6 (0.9)	7.6 (3.5)		0 (0)	0.5 (0)	1.7 (1.0)	7.5 (3.9)		0 (0)	0.5 (0)	1.7 (1.0)	7.7 (4.1)
Milk chocolate	0.6 (1.5)	0.7 (1.2)	1.1 (1.7)	1.6 (3)		0.7 (1.5)	0.8 (1.4)	1.2 (1.7)	1.5 (2.6)		0.5 (1.4)	0.8 (1.4)	1.5 (1.9)	1.9 (3.3)
Total chocolate†	0.6 (1.5)	1.2 (1.2)	2.7 (2.0)	9.2 (4.8)		0.7 (1.5)	1.3 (1.4)	2.9 (2.0)	9.0 (4.7)		0.5 (1.4)	1.3 (1.4)	3.2 (2.2)	9.6 (5.5)
Age (years)	71.3 (6.7)	68.7 (6.7)	68.9 (6.7)	67.3 (6.2)		52.0 (4.7)	52.4 (4.6)	52.5 (4.5)	52.9 (4.5)		68.4 (7.3)	68.2 (7.6)	68.3 (7.6)	68.5 (7.6)
White (% (No))	97.7 (24 889)	97.8 (7421)	98.4 (5279)	98.9 (959)		95.8 (26 327)	96.5 (15 540)	97.0 (11 584)	97.7 (2613)		95.5 (7064)	96.3 (3451)	96.4 (2464)	97.1 (519)
Current smoking (% (No))	6.7 (1714)	6.0 (453)	5.7 (307)	6.1 (59)		7.1 (1945)	5.9 (948)	5.4 (639)	4.4 (118)		3.4 (248)	2.7 (97)	2.6 (67)	3.3 (18)
Alcohol intake (g/day)	6.7 (11.4)	6.7 (10.7)	6.7 (10.2)	6.4 (10)		6.4 (10.9)	6.8 (10)	7.1 (10.2)	6.7 (9.7)		14.4 (17.6)	13.8 (15.6)	13.3 (14.9)	13.9 (14.4)
Median (SD) physical activity (MET-h/week)	21.7 (26.1)	23.7 (26.8)	23.8 (26.5)	24.8 (28.4)		22.6 (29.4)	23.5 (29.1)	24.8 (28.2)	26.9 (28.2)		41.1 (34.4)	42.8 (36.3)	42.6 (35.1)	45.9 (36.1)
BMI	26.2 (5.1)	26.2 (4.9)	26.1 (4.9)	25.6 (4.5)		27.2 (6.2)	27.0 (5.9)	26.7 (5.8)	25.6 (5.3)		26.0 (3.7)	26.1 (3.5)	25.8 (3.4)	25.1 (3.1)
Family history of diabetes (% (No))	23.1 (5877)	22.4 (1702)	22.2 (1193)	22.3 (216)		15.0 (4130)	14.6 (2354)	14.3 (1710)	12.4 (331)		17.5 (1294)	18.6 (668)	17.3 (442)	17.1 (91)
Multivitamin use (% (No))	71.2 (18 132)	74.9 (5683)	76.0 (4077)	78.7 (763)		56.1 (15 422)	59.8 (9629)	61.3 (7326)	66.4 (1777)		67.6 (5001)	69.4 (2486)	70.1 (1791)	78.1 (418)
Use of any postmenopausal hormone (% (No))	74.9 (19 087)	75.6 (5738)	75.8 (4068)	76.5 (742)		41.3 (11 358)	41.0 (6596)	39.9 (4766)	39.6 (1058)		–	–	–	–
Use of oral contraceptives (% (No))	–	–	–	–		12.5 (3433)	12.5 (2017)	13.2 (1582)	13.4 (357)		–	–	–	–
Baseline hypertension (% (No))	57.7 (14 701)	55.8 (4236)	56.2 (3012)	56.8 (551)		27.9 (7662)	26.6 (4278)	25.2 (3011)	22.3 (596)		46.8 (3462)	44.4 (1592)	43.8 (1119)	42.2 (226)
Baseline hypocholesterolemia (% (No))	68.0 (17 312)	68.9 (5228)	68.5 (3674)	71.0 (689)		41.9 (11 514)	42.3 (6814)	41.8 (4998)	40.2 (1076)		56.8 (4203)	57.7 (2068)	57.8 (1477)	54.1 (289)
Total energy intake (kcal/day)	1589 (509)	1743 (520)	1886 (542)	2138 (563)		1687 (534)	1830 (539)	1991 (540)	2221 (545)		1927 (600)	2078 (614)	2244 (639)	2515 (655)
AHEI	59.3 (12.1)	61.2 (12.2)	61.6 (12.5)	61.4 (12.4)		60.8 (12.7)	63.4 (12.6)	64.2 (12.6)	65.2 (13.1)		62.2 (12.0)	62.4 (11.7)	63.1 (12.0)	65.0 (12.6)
Red or processed meats (servings/day)	0.7 (0.5)	0.7 (0.5)	0.8 (0.5)	0.7 (0.5)		0.8 (0.6)	0.9 (0.6)	0.9 (0.6)	0.8 (0.6)		1.0 (0.7)	1.1 (0.7)	1.1 (0.8)	0.9 (0.7)
Fruit and vegetables (servings/day)	4.8 (2.6)	5.2 (2.6)	5.4 (2.7)	5.5 (2.7)		4.9 (2.9)	5.4 (2.9)	5.6 (2.9)	5.8 (3.1)		5.7 (2.3)	6.0 (2.3)	6.3 (2.5)	6.6 (2.5)
Whole grain (servings/day)	1.3 (1.0)	1.3 (1.0)	1.4 (1.0)	1.4 (1.0)		1.1 (1.0)	1.3 (0.9)	1.3 (1.0)	1.4 (1.0)		0.7 (0.7)	0.7 (0.7)	0.7 (0.6)	0.8 (0.6)
Sugar sweetened beverages (servings/day)	0.2 (0.5)	0.2 (0.4)	0.2 (0.4)	0.2 (0.4)		0.3 (0.7)	0.2 (0.5)	0.2 (0.5)	0.2 (0.5)		0.2 (0.4)	0.2 (0.4)	0.2 (0.4)	0.2 (0.4)
Tea (servings/day)	0.4 (0.8)	0.5 (0.9)	0.5 (0.9)	0.5 (1.0)		0.8 (1.3)	0.9 (1.3)	1.0 (1.3)	1.1 (1.4)		0.5 (0.9)	0.5 (0.9)	0.6 (0.9)	0.8 (1.1)
Coffee (servings/day)	1.2 (1.3)	1.2 (1.3)	1.1 (1.3)	1 (1.3)		1.2 (1.4)	1.3 (1.4)	1.2 (1.3)	1.1 (1.3)		1.6 (1.5)	1.6 (1.5)	1.5 (1.5)	1.6 (1.5)
Sweets and desserts (servings/day)	0.9 (1.1)	1.1 (1.1)	1.5 (1.2)	2.5 (1.6)		0.7 (0.7)	0.8 (0.7)	1.1 (0.8)	2.1 (1.1)		0.9 (1.0)	1.1 (1.0)	1.6 (1.1)	2.5 (1.6)
Saturated fat (% energy)	9.7 (2.9)	10.1 (2.6)	10.6 (2.6)	12.7 (2.8)		10.1 (2.7)	10.3 (2.4)	10.9 (2.4)	12.6 (2.6)		9.5 (2.7)	10.0 (2.5)	10.7 (2.5)	12.0 (2.7)
Added sugar (g/day)	38.3 (21.8)	38.9 (19.2)	41.5 (18.1)	49.3 (18.2)		43.7 (27.4)	42.3 (22.3)	45.1 (20.2)	52.9 (19.8)		44.8 (26.7)	47.6 (22.8)	51.3 (23.2)	58.1 (22.9)
Cereal fiber (g/day)	6.6 (3.2)	6.4 (2.8)	6.3 (2.9)	5.4 (2.4)		7.0 (3.6)	6.9 (3.2)	6.8 (3.0)	6.2 (3.0)		7.4 (3.8)	7.3 (3.5)	6.8 (2.9)	6.2 (2.7)
Epicatechin (mg/day)	9.0 (6.9)	10.4 (6.3)	12.4 (6.2)	22.1 (8.7)		10.1 (8.0)	11.8 (7.3)	14.1 (7.2)	25.2 (10.0)		10.6 (7.6)	11.9 (6.7)	14.5 (7.3)	26.4 (10.2)
Total flavonoids (mg/day)	355.4 (296.9)	369.8 (274.4)	373.9 (252.3)	412.2 (266.9)		397.4 (362.1)	413.9 (322.2)	423.5 (299.7)	481.6 (303.7)		382.2 (279.3)	387.3 (245.3)	407.7 (254.7)	497.2 (270.6)

1 kcal=4.18 kJ=0.00418 MJ.

AHEI=Alternate Healthy Eating Index; BMI=body mass index; NHS=Nurses’ Health Study; NHSII=Nurses’ Health Study II; HPFS=Health Professionals Follow-up Study; MET-h=metabolic equivalent tasks per hour; SD=standard deviation.

* Baseline years were 2006 for NHS, 2007 for NHSII, and 2006 for HPFS.

† Cumulative averages of total chocolate intake in 1980-2006 (NHS), 1991-2007 (NHSII), and 1986-2006 (HPFS).

**Table 2:** Age standardized baseline characteristics according to milk chocolate* intake at baseline. Values are mean (SD) unless stated otherwise

	Frequency of milk chocolate intake													
	NHS					NHSII					HPFS			
	0 or <1 serving/month	1 serving/month to <1 serving/week	1-4 servings/week	≥5 servings/week		0 or <1 serving/month	1 serving/month to <1 serving/week	1-4 servings/week	≥5 servings/week		Never or <1 serving/month	1 serving/month to <1 serving/week	1-4 servings/week	≥5 servings/week
No of participants	19 327	11 269	7688	1116		20 822	20 380	14 901	2084		5520	4611	3457	479
Milk chocolate (servings/week)	0 (0)	0.5 (0)	1.7 (1)	7.5 (3.9)		0 (0)	0.5 (0)	1.7 (1.0)	7.2 (3.6)		0 (0)	0.5 (0)	1.7 (1.0)	7.3 (3.5)
Dark chocolate (servings/week)	0.4 (1.6)	0.4 (1.1)	0.7 (1.4)	1.3 (2.7)		0.7 (1.9)	0.7 (1.6)	1.0 (1.8)	1.9 (3)		0.5 (1.7)	0.6 (1.6)	1.0 (1.6)	2.2 (3.4)
Total chocolate† (servings/week)	0.4 (1.6)	0.9 (1.1)	2.4 (1.8)	8.9 (4.8)		0.7 (1.9)	1.2 (1.6)	2.7 (2.1)	9.1 (4.7)		0.5 (1.7)	1.1 (1.6)	2.7 (2.0)	9.6 (5.3)
Age (years)	71.3 (6.6)	69.2 (6.6)	69.7 (6.9)	70.5 (7.3)		52.8 (4.6)	52.1 (4.7)	51.9 (4.6)	52 (4.6)		68.8 (7.5)	68.0 (7.4)	67.9 (7.5)	68.5 (7.8)
White (% (No))	97.7 (18 884)	97.6 (11004)	98.4 (7562)	98.4 (1098)		95.6 (19 912)	96.3 (19 620)	97.3 (14 497)	98.0 (2043)		95.3 (5262)	96.2 (4437)	96.3 (3328)	97.1 (465)
Current smoking (% (No))	5.8 (1114)	6.2 (701)	7.6 (586)	10.5 (117)		5.8 (1210)	6.4 (1312)	6.7 (991)	7.5 (156)		2.8 (155)	2.9 (134)	3.4 (117)	6.3 (30)
Alcohol intake (g/day)	7.7 (12.1)	6.2 (10.4)	5.2 (9.2)	4.4 (8.6)		7.9 (11.9)	6.5 (9.9)	5.5 (8.9)	4.3 (7.8)		15.7 (18.0)	13.6 (15.7)	12.4 (15.1)	10.7 (15.1)
Median (SD) physical activity (MET-h/week)	23.9 (27.9)	21.2 (24.4)	20.8 (25.3)	20.3 (25.4)		26.1 (31.8)	22.4 (27.0)	21.8 (27.8)	20.8 (26.5)		42.2 (35.1)	42 (34.6)	41.8 (35.7)	41.1 (35.1)
BMI	25.7 (4.9)	26.5 (5.0)	26.6 (5.1)	26.2 (5.3)		26.2 (5.6)	27.1 (6.0)	27.6 (6.4)	27.7 (6.5)		25.6 (3.5)	26.2 (3.6)	26.3 (3.6)	26.2 (3.2)
Family history of diabetes (% (No))	22.3 (4304)	23.2 (2616)	23.8 (1828)	21.9 (244)		14.3 (2986)	15.0 (3051)	14.7 (2196)	13.9 (290)		17.6 (973)	17.9 (827)	17.5 (604)	16.5 (79)
Multivitamin use (% (No))	73.5 (14 215)	72.8 (8202)	71.1 (5470)	69.9 (780)		59.8 (12 447)	58.9 (11 997)	57.0 (8487)	57.7 (1203)		69.8 (3853)	68.6 (3162)	68.3 (2362)	67.1 (321)
Use of any postmenopausal hormone (% (No))	75.1 (14 521)	75.0 (8457)	74.7 (5742)	75.7 (845)		41.1 (8559)	41.0 (8349)	40.4 (6015)	41.8 (872)		–	–	–	–
Use of oral contraceptives (% (No))	–	–	–	–		12.2 (2537)	12.5 (2544)	13.5 (2016)	13.8 (287)		–	–	–	–
Baseline hypertension (% (No))	56.4 (10 909)	58.0 (6534)	56.9 (4378)	59.9 (668)		25.4 (5296)	27.3 (5560)	27.8 (4135)	27.8 (578)		45.8 (2530)	45.6 (2104)	45.0 (1557)	47.7 (228)
Baseline hypocholesterolemia (% (No))	66.7 (12 889)	69.6 (7844)	69.8 (5369)	71.6 (799)		40.4 (8406)	42.4 (8645)	43.0 (6412)	46.3 (964)		56.4 (3111)	57.2 (2638)	58.3 (2016)	56.8 (272)
Total energy intake (kcal/day)	1564 (501)	1696 (520)	1849 (548)	2099 (568)		1674 (529)	1806 (535)	1961 (557)	2211 (572)		1880 (579)	2041 (603)	2244 (646)	2560 (679)
AHEI	62.1 (12.5)	59.2 (11.8)	57.1 (11.6)	54.4 (11.4)		65.7 (12.8)	62.0 (12.3)	59.3 (12.2)	56.5 (12.1)		64.9 (12.2)	62.1 (11.6)	59.9 (11.3)	57.0 (11.2)
Red or processed meats (servings/day)	0.7 (0.5)	0.8 (0.5)	0.8 (0.6)	0.9 (0.6)		0.7 (0.6)	0.9 (0.6)	1.0 (0.6)	1.0 (0.6)		0.9 (0.7)	1.1 (0.7)	1.2 (0.8)	1.2 (0.8)
Fruit and vegetables (servings/day)	5.1 (2.7)	4.9 (2.5)	4.9 (2.5)	4.6 (2.5)		5.5 (3.2)	5.2 (2.8)	5.0 (2.7)	4.7 (2.6)		6.1 (2.5)	5.8 (2.3)	5.9 (2.3)	5.6 (2.2)
Whole grain (servings/day)	1.3 (1.0)	1.3 (1.0)	1.3 (1.0)	1.2 (1.0)		1.2 (1.0)	1.2 (0.9)	1.2 (0.9)	1.2 (1.0)		0.7 (0.7)	0.7 (0.6)	0.7 (0.6)	0.7 (0.6)
Sugar sweetened beverages (servings/day)	0.1 (0.4)	0.2 (0.4)	0.2 (0.5)	0.3 (0.7)		0.2 (0.5)	0.2 (0.6)	0.3 (0.7)	0.3 (0.8)		0.1 (0.4)	0.2 (0.4)	0.2 (0.5)	0.3 (0.5)
Tea (servings/day)	0.4 (0.8)	0.4 (0.8)	0.4 (0.8)	0.5 (0.9)		0.9 (1.3)	0.8 (1.2)	0.9 (1.2)	0.9 (1.3)		0.6 (1.0)	0.5 (0.9)	0.5 (0.9)	0.5 (0.9)
Coffee (servings/day)	1.2 (1.3)	1.2 (1.3)	1.1 (1.3)	1.1 (1.4)		1.3 (1.3)	1.3 (1.4)	1.1 (1.4)	1.0 (1.4)		1.6 (1.5)	1.6 (1.5)	1.6 (1.5)	1.4 (1.6)
Sweets and desserts (servings/day)	0.8 (0.9)	1.1 (1.0)	1.6 (1.2)	2.9 (1.6)		0.5 (0.6)	0.8 (0.6)	1.2 (0.8)	2.4 (1.3)		0.7 (0.9)	1.1 (0.9)	1.6 (1.1)	3.0 (1.7)
Saturated fat (% energy)	9.3 (2.8)	10.2 (2.6)	11 (2.5)	13.5 (2.8)		9.7 (2.7)	10.4 (2.4)	11.1 (2.3)	13.4 (2.5)		9.1 (2.7)	10.0 (2.5)	10.8 (2.3)	12.9 (2.4)
Added sugar (g/day)	34.8 (20.4)	40.5 (19.8)	45.5 (20.2)	57 (20.4)		38.2 (24.3)	43.8 (23.3)	50.0 (23.8)	61.9 (23.2)		40.2 (24.8)	47.4 (22.9)	55.2 (24.5)	68.2 (25.5)
Cereal fiber (g/day)	6.7 (3.3)	6.4 (2.9)	6.1 (2.6)	5.3 (2.6)		7.1 (3.7)	7.0 (3.1)	6.6 (3)	5.8 (2.7)		7.6 (3.9)	7.2 (3.3)	6.9 (3.1)	6.0 (2.7)
Epicatechin (mg/day)	10.5 (7.7)	9.5 (6.5)	9.6 (6.2)	11.0 (7.1)		13 (9.6)	11.4 (7.7)	11.5 (7.4)	13.3 (8.4)		12.9 (9.1)	11.7 (7.4)	11.8 (7.1)	14.2 (8.3)
Total flavonoids (mg/day)	382.4 (305.2)	349.3 (275.6)	334.1 (254.9)	343.8 (259.4)		453.4 (379.4)	395.1 (314.0)	377.3 (301.0)	379.6 (284.0)		426.4 (300.7)	376.9 (241.8)	362.4 (245.2)	369.3 (212.3)

1 kcal=4.18 kJ=0.00418 MJ.

AHEI=Alternate Healthy Eating Index; BMI=body mass index; NHS=Nurses’ Health Study; NHSII=Nurses’ Health Study II; HPFS=Health Professionals Follow-up Study; MET-h=metabolic equivalent tasks per hour; SD=standard deviation.

* Baseline years were 2006 for NHS, 2007 for NHSII, and 2006 for HPFS.

† Cumulative averages of total chocolate intake in 1980-2006 (NHS), 1991-2007 (NHSII), and 1986-2006 (HPFS).

When examining the crude associations between chocolate consumption and selected nutrients, foods, and drinks at study baseline, milk chocolate consumption showed stronger positive associations with the consumption of less healthy food and nutrients, including saturated fat, added sugar, red and processed meat, and sweets and desserts. Dark chocolate consumption was positively associated with intakes of flavan-3-ols, particularly epicatechin. Overall, dark chocolate consumption was more positively associated with intakes of other flavan-3-ols-rich food and beverages, such as blueberries, tea, and red wine. The β coefficients between total chocolate consumption and these nutrients or food and beverages were similar to the coefficients observed for milk chocolate (data not shown).

### Chocolate intake and T2D

In the primary analyses for total chocolate, 18 862 people with incident T2D were identified during 4 829 175 person years of follow-up. In the age and calendar time stratified Cox proportional hazards models, no significant associations were observed between total chocolate consumption and risk of T2D in the pooled dataset (table 3). After adjusting for lifestyle and dietary risk factors, we found that participants who consumed ≥5 servings/week of any chocolate showed a 10% (95% CI 2% to 17%; P trend=0.07) lower relative risk of T2D compared with those who never or rarely consumed chocolate. A marginally significant 1% (0% to 2%) reduction in risk of T2D was observed for each serving/week consumption of total chocolate.

**Table 3:** Adjusted hazard ratios (95% CIs)* of T2D for total chocolate intake in NHS (1986-2018), NHSII (1991-2021), and HPFS (1986-2020)

	Total chocolate consumption levels				P trend†	Per serving/week
	0 or <1 serving/month	1 serving/month to <1 serving/week	1-4 servings/week	≥5 servings/week		
**NHS**						
Case/person years	2287/579 899	3254/682 452	1541/305 768	169/35 041		
Age adjusted	1	1.22 (1.16 to 1.29)	1.32 (1.23 to 1.41)	1.30 (1.11 to 1.53)	<0.001	1.04 (1.02 to 1.05)
Multivariable adjusted	1	1.05 (0.99 to 1.11)	1.05 (0.98 to 1.12)	0.98 (0.83 to 1.15)	0.76	1.00 (0.98 to 1.02)
**NHSII**						
Case/person years	1084/416 282	3391/1 021 600	2740/725 061	350/105 441		
Age adjusted	1	1.21 (1.13 to 1.30)	1.38 (1.28 to 1.48)	1.27 (1.12 to 1.43)	<0.001	1.05 (1.03 to 1.07)
Multivariable adjusted	1	1.00 (0.94 to 1.08)	0.99 (0.92 to 1.06)	0.84 (0.74 to 0.95)	0.02	0.99 (0.97 to 1.00)
**HPFS**						
Case/person years	1146/302 010	1742/390 683	1026/232 552	132/32 386		
Age adjusted	1	1.19 (1.11 to 1.29)	1.21 (1.11 to 1.32)	1.15 (0.96 to 1.38)	0.008	1.01 (0.99 to 1.03)
Multivariable adjusted	1	1.04 (0.96 to 1.13)	1.04 (0.95 to 1.14)	0.92 (0.76 to 1.11)	0.65	0.98 (0.96 to 1.01)
**Pooled‡**						
Case/person years	4520/1 299 997	8395/2 097 808	5309/1 264 790	651/173 110		
Age adjusted	1	1.21 (1.16 to 1.25)	1.32 (1.27 to 1.38)	1.24 (1.14 to 1.35)	<0.001	1.03 (1.02 to 1.04)
Multivariable adjusted	1	1.03 (1.00 to 1.07)	1.02 (0.98 to 1.07)	0.90 (0.83 to 0.98)	0.07	0.99 (0.98 to 1.00)

Multivariable models adjusted for age, calendar year, ethnicity (white, African American, Asian, and others), smoking status (never, former, current (1-14, 15-24, or ≥25 cigarettes/day), or missing), alcohol intake (g/day: 0, 0.1-4.9, 5.0-14.9, and ≥15.0 in women, 0, 0.1-4.9, 5.0-14.9, 15.0-29.9, and ≥30.0 in men, or missing), family history of diabetes (yes/no), menopausal status and postmenopausal hormone use (premenopausal, postmenopausal (never, former, or current hormone use), or missing, for women), use of oral contraceptives (yes, no, NHSII only), physical activity (<3, 3.0-8.9, 9.0-17.9, 18.0-26.9, ≥27.0 MET-h/week, or missing), baseline BMI (<21.0, 21.0-22.9, 23.0-24.9, 25.0-26.9, 27.0-29.9, 30.0-32.9, 33.0-34.9, ≥35.0, or missing), multivitamin use (yes/no), baseline hypertension, baseline hypercholesterolemia, total energy intake, and AHEI (five groups).

AHEI=Alternate Healthy Eating Index; BMI=body mass index; CI=confidence interval; NHS=Nurses’ Health Study; NHSII=Nurses’ Health Study II; HPFS=Health Professionals Follow-up Study; MET-h= metabolic equivalent tasks per hour; T2D=type 2 diabetes.

* Calculated using Cox proportional hazards models.

† Calculated using median levels of chocolate consumption categories as continuous predictor in the model.

‡ Data from three cohorts were combined to run the pooled results. Pooled models further adjusted for study origin (NHS, NHSII, HPFS).

For analyses by chocolate subtypes, 4771 people with incident T2D were identified during 1 270 348 person years of follow-up (table 4). After adjusting for confounding factors, participants who consumed ≥5 servings/week of dark chocolate had a significant 21% (5% to 34%) lower rate of T2D compared with those who never or rarely ate dark chocolate, and a significant linear trend across four groups was observed (P trend=0.006). A significant 3% (1% to 5%) reduction in risk of T2D was observed for each serving/week consumption of dark chocolate.

**Table 4:** Adjusted hazard ratios (95% CIs)* of T2D for chocolate intake by subtype in NHS (2006-18), NHSII (2007-21), and HPFS (2006-20)

	Chocolate subtypes consumption levels				P trend†	Per serving/week
	0 or <1 serving/month	1 serving/month to <1 serving/week	1-4 servings/week	≥5 servings/week		
**Dark chocolate**						
NHS:						
Case/person years	789/229 604	300/104 135	174/51 245	32/10 517		
Age adjusted	1	0.91 (0.79 to 1.04)	0.94 (0.80 to 1.11)	0.90 (0.63 to 1.29)	0.42	0.98 (0.94 to 1.02)
Multivariable adjusted	1	0.92 (0.80 to 1.06)	0.99 (0.83 to 1.17)	0.99 (0.68 to 1.43)	0.92	0.99 (0.95 to 1.04)
NHSII:						
Case/person years	1339/291 824	971/241 896	505/145 064	88/35 192		
Age adjusted	1	0.87 (0.80 to 0.94)	0.75 (0.67 to 0.83)	0.53 (0.43 to 0.66)	<0.001	0.91 (0.88 to 0.93)
Multivariable adjusted	1	0.95 (0.87 to 1.03)	0.91 (0.82 to 1.02)	0.80 (0.64 to 1.01)	0.03	0.97 (0.94 to 1.00)
HPFS:						
Case/person years	263/70 984	202/52 587	97/30 207	11/7093		
Age adjusted	1	1.09 (0.91 to 1.32)	0.90 (0.71 to 1.14)	0.44 (0.24 to 0.80)	0.006	0.90 (0.84 to 0.97)
Multivariable adjusted	1	1.08 (0.89 to 1.31)	0.95 (0.74 to 1.22)	0.49 (0.26 to 0.92)	0.04	0.92 (0.85 to 0.99)
Pooled‡:						
Case/person years	2404/594 879	1481/399 934	777/227 163	132/52 895		
Age adjusted	1	0.91 (0.85 to 0.97)	0.81 (0.74 to 0.88)	0.58 (0.49 to 0.70)	<0.001	0.92 (0.90 to 0.95)
Multivariable adjusted	1	0.95 (0.89 to 1.02)	0.93 (0.85 to 1.01)	0.79 (0.66 to 0.95)	0.006	0.97 (0.95 to 0.99)
**Milk chocolate**						
NHS:						
Case/person years	530/183 122	445/135 119	280/66 828	40/10 432		
Age adjusted	1	1.12 (0.99 to 1.28)	1.30 (1.12 to 1.51)	1.29 (0.93 to 1.78)	0.002	1.03 (0.99 to 1.06)
Multivariable adjusted	1	0.95 (0.83 to 1.08)	1.05 (0.90 to 1.23)	1.02 (0.72 to 1.44)	0.52	1.00 (0.96 to 1.04)
NHSII:						
Case/person years	815/236 945	1229/295 724	752/158 452	107/22 855		
Age adjusted	1	1.24 (1.13 to 1.36)	1.43 (1.29 to 1.58)	1.41 (1.15 to 1.72)	<0.001	1.05 (1.03 to 1.07)
Multivariable adjusted	1	0.96 (0.88 to 1.05)	0.97 (0.87 to 1.08)	0.90 (0.72 to 1.12)	0.49	0.98 (0.96 to 1.01)
HPFS:						
Case/person years	180/56 529	235/63 694	136/35 662	22/4988		
Age adjusted	1	1.24 (1.02 to 1.51)	1.21 (0.96 to 1.51)	1.32 (0.84 to 2.08)	0.17	1.03 (0.98 to 1.08)
Multivariable adjusted	1	1.05 (0.86 to 1.29)	0.95 (0.74 to 1.22)	1.00 (0.61 to 1.63)	0.69	0.99 (0.93 to 1.05)
Pooled‡:						
Case/person years	1533/478 373	1918/496 219	1177/261 872	169/38 409		
Age adjusted	1	1.21 (1.13 to 1.29)	1.36 (1.26 to 1.47)	1.36 (1.16 to 1.59)	<0.001	1.04 (1.02 to 1.06)
Multivariable adjusted	1	0.97 (0.90 to 1.04)	0.99 (0.91 to 1.08)	0.94 (0.79 to 1.12)	0.75	0.99 (0.97 to 1.01)

Multivariable models adjusted for age, calendar year, ethnicity (white, African American, Asian, and others), smoking status (never, former, current (1-14, 15-24, or ≥25 cigarettes/day), or missing), alcohol intake (g/day: 0, 0.1-4.9, 5.0-14.9, and ≥15.0 in women, 0, 0.1-4.9, 5.0-14.9, 15.0-29.9, and ≥30.0 in men, or missing), family history of diabetes (yes/no), menopausal status and postmenopausal hormone use (premenopausal, postmenopausal (never, former, or current hormone use), or missing, for women), use of oral contraceptives (yes, no, NHSII only), physical activity (<3, 3.0-8.9, 9.0-17.9, 18.0-26.9, ≥27.0 MET-h/week, or missing), baseline BMI (<21.0, 21.0-22.9, 23.0-24.9, 25.0-26.9, 27.0-29.9, 30.0-32.9, 33.0-34.9, ≥35.0, or missing), multivitamin use (yes/no), baseline hypertension, baseline hypercholesterolemia, total energy intake, AHEI (five groups), and total chocolate intake before baseline (as cumulative averages for 1980-2002 (NHS), 1991-2003 (NHSII), 1986-2002 (HPFS)).

AHEI=Alternate Healthy Eating Index; BMI=body mass index; CI=confidence interval; NHS=Nurses’ Health Study; NHSII=Nurses’ Health Study II; HPFS=Health Professionals Follow-up Study; MET-h=metabolic equivalent tasks per hour; T2D=type 2 diabetes.

* Calculated using Cox proportional hazards models.

† Calculated using median levels of chocolate consumption categories as continuous predictor in the model.

‡ Data from three cohorts were combined to run the pooled results. Pooled models further adjusted for study origin (NHS, NHSII, HPFS).

Associations between milk chocolate intake and risk of T2D were largely null. The multivariable adjusted hazard ratio for consumption of milk chocolate and risk of T2D was 0.94 (95% CI 0.79 to 1.12; P trend=0.75), comparing extreme consumption groups (high versus low).

Statistically significant heterogeneity was observed when examining results across the three cohorts. In the analysis of total chocolate, stronger associations were observed in NHSII, with consumption of ≥5 servings/week of total chocolate associated with a 16% (5% to 26%) lower risk of T2D compared with those who rarely consumed chocolate. In the analyses by chocolate subtypes, HPFS showed the most significant associations for dark chocolate consumption, with consumption of ≥5 servings/week of dark chocolate associated with a 51% (8% to 74%) lower risk of T2D compared with those who never or rarely consumed dark chocolate. Similar associations and trends were observed in NHSII, but the results were less significant. In NHS, neither total nor subtypes of chocolate consumption were statistically significantly associated with risk of T2D.

**Fig 1:**
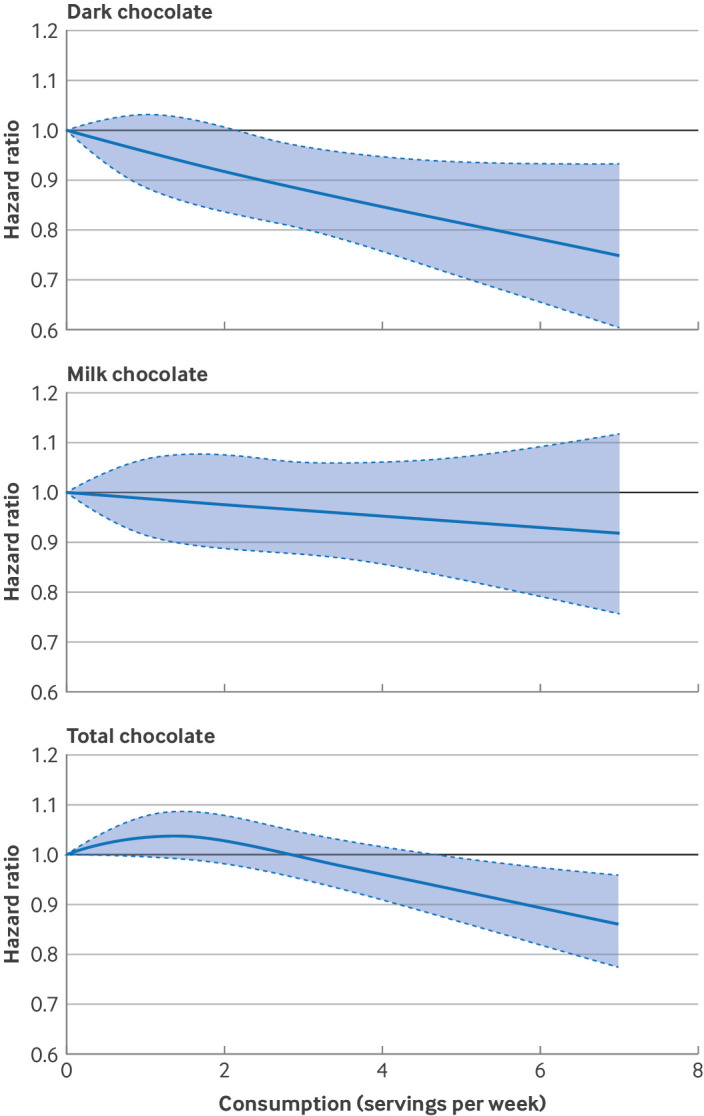
Multivariable adjusted, pooled, dose-response associations between chocolate intake and risk of type 2 diabetes in NHS, NHSII, and HPFS. Dose-response analysis was performed using SAS Macro %LGTPHCURV9 (number of knots=3). Data from three cohorts were combined and truncated at the first and 99th centiles. P values for linearity and non-linearity were, respectively, P=0.003 and P=0.96 for dark chocolate, P=0.39 and P=0.99 for milk chocolate, and P=0.08 and P=0.008 for total chocolate. P values for non-linearity were obtained as the significance of the spline terms, and P values for linearity were obtained as the significance of the linear terms. P<0.05 indicates statistical significance. For dark and milk chocolate, follow-up periods were 2006-18 for NHS, 2007-21 for NHSII, and 2006-20 for HPFS. For total chocolate, follow-up periods were 1986-2018 for NHS, 1991-2021 for NHSII, and 1986-2020 for HPFS. Hazard ratios were adjusted for age, calendar year, ethnicity (white, African American, Asian, and others), smoking status (never, former, current (1-14, 15-24, or ≥25 cigarettes/day), or missing), alcohol intake (g/day: 0, 0.1-4.9, 5.0-14.9, and ≥15.0 in women, 0, 0.1-4.9, 5.0-14.9, 15.0-29.9, and ≥30.0 in men, or missing), family history of diabetes (yes/no), menopausal status and postmenopausal hormone use (premenopausal, postmenopausal (never, former, or current hormone use), or missing, women only), use of oral contraceptives (yes, no, NHSII only), physical activity (<3, 3.0-8.9, 9.0-17.9, 18.0-26.9, ≥27.0 MET-h/week, or missing), BMI (<21.0, 21.0-22.9, 23.0-24.9, 25.0-26.9, 27.0-29.9, 30.0-32.9, 33.0-34.9, ≥35.0, or missing), multivitamin use (yes/no), baseline hypertension, baseline hypercholesterolemia, total energy intake, AHEI (five groups), and study origin (NHS, NHSII, HPFS). For dark and milk chocolate dose-response analysis, total chocolate intake before baseline (as cumulative averages 1980-2002 for NHS, 1991-2003 for NHSII, and 1986-2002 for HPFS) were further adjusted. AHEI=Alternate Healthy Eating Index; BMI=body mass index; NHS=Nurses’ Health Study; NHSII=Nurses’ Health Study II; HPFS=Health Professionals Follow-up Study; MET-h=metabolic equivalent tasks per hour

Restricted cubic spline regression revealed a linear dose-response association between dark chocolate intake and risk of T2D (P for linearity=0.003; fig 1). The association between total chocolate intake and risk of T2D appeared to be non-linear (P for non-linearity=0.008). The dose-response association between milk chocolate intake with risk of T2D remained essentially null. The results continued to be robust without excluding outliers (supplementary figure S2).

### Subgroup analyses

In stratified analyses, significant effect modifications were observed for overall dietary quality and associations of dark chocolate with risk of T2D (P for interaction=0.007; table 5). Specifically, participants with a higher quality diet, measured by AHEI (≥median), showed stronger associations of dark chocolate consumption with risk of T2D (34% (95% CI 12% to 51%) lower risk of T2D comparing ≥5 serving/week to reference group) than individuals with a lower quality diet (hazard ratio 0.87, 95% CI 0.68 to 1.10, comparing extreme groups). Among those younger than 70 years, male participants, those with higher physical activity, and those with no family history of diabetes, the risk of T2D was lower when comparing extreme groups of dark chocolate intake, although P values for interactions were not statistically significant. Age, sex, BMI, physical activity, AHEI, and family history of diabetes did not seem to significantly modify the associations of total and milk chocolate intake with risk of T2D.

**Table 5:** Stratified analyses by selected characteristics. Values are hazard ratio (95% CI)* unless stated otherwise

	Chocolate consumption levels				P trend†	P for interaction†
	0 or <1 serving/month	1 serving/month to <1 serving/week	1-4 servings/week	≥5 servings/week		
**Total chocolate‡**						
Age (years):						
<70	1	1.02 (0.97 to 1.06)	1.02 (0.97 to 1.07)	0.88 (0.80 to 0.97)	0.07	0.10
≥70	1	1.11 (1.03 to 1.20)	1.01 (0.92 to 1.12)	1.01 (0.81 to 1.28)	0.61	
Sex:						
Male	1	1.04 (0.96 to 1.13)	1.04 (0.95 to 1.14)	0.92 (0.76 to 1.11)	0.64	0.92
Female	1	1.03 (0.99 to 1.08)	1.02 (0.97 to 1.07)	0.90 (0.81 to 0.99)	0.08	
BMI:						
<30	1	1.05 (0.99 to 1.11)	1.03 (0.97 to 1.10)	0.97 (0.84 to 1.11)	0.76	0.31
≥30	1	1.03 (0.98 to 1.09)	0.99 (0.94 to 1.06)	0.84 (0.75 to 0.94)	0.002	
Physical activity§:						
<Median	1	1.05 (1.00 to 1.10)	1.04 (0.99 to 1.10)	0.92 (0.83 to 1.02)	0.32	0.51
≥Median	1	1.02 (0.96 to 1.08)	0.99 (0.92 to 1.06)	0.89 (0.77 to 1.04)	0.11	
AHEI§:						
<Median	1	1.02 (0.97 to 1.08)	1.02 (0.96 to 1.08)	0.94 (0.85 to 1.05)	0.36	0.25
≥Median	1	1.06 (1.01 to 1.13)	1.06 (0.99 to 1.14)	0.82 (0.70 to 0.97)	0.35	
Family history of diabetes:						
No	1	1.03 (0.98 to 1.08)	1.02 (0.97 to 1.08)	0.86 (0.78 to 0.96)	0.048	0.20
Yes	1	1.03 (0.97 to 1.10)	1.01 (0.94 to 1.09)	0.96 (0.83 to 1.12)	0.55	
**Dark chocolate**¶						
Age (years):						
<70	1	0.96 (0.89 to 1.03)	0.91 (0.83 to 1.00)	0.77 (0.63 to 0.94)	0.004	0.74
≥70	1	0.95 (0.81 to 1.11)	1.02 (0.83 to 1.24)	0.96 (0.61 to 1.49)	0.99	
Sex:						
Male	1	1.08 (0.89 to 1.31)	0.95 (0.74 to 1.22)	0.49 (0.26 to 0.92)	0.04	0.17
Female	1	0.94 (0.87 to 1.01)	0.92 (0.84 to 1.01)	0.83 (0.69 to 1.01)	0.03	
BMI:						
<30	1	0.98 (0.87 to 1.09)	0.93 (0.81 to 1.07)	0.80 (0.61 to 1.06)	0.09	0.94
≥30	1	0.95 (0.87 to 1.04)	0.91 (0.81 to 1.02)	0.81 (0.63 to 1.04)	0.04	
Physical activity§:						
<Median	1	0.97 (0.89 to 1.06)	0.93 (0.84 to 1.04)	0.88 (0.70 to 1.11)	0.14	0.24
≥Median	1	0.92 (0.82 to 1.04)	0.90 (0.78 to 1.04)	0.63 (0.46 to 0.87)	0.005	
AHEI§:						
<Median	1	1.04 (0.95 to 1.13)	0.94 (0.83 to 1.05)	0.87 (0.68 to 1.10)	0.10	0.007
≥Median	1	0.82 (0.73 to 0.92)	0.88 (0.77 to 1.01)	0.66 (0.49 to 0.88)	0.009	
Family history of diabetes:						
No	1	0.96 (0.88 to 1.04)	0.96 (0.86 to 1.06)	0.76 (0.61 to 0.95)	0.03	0.64
Yes	1	0.90 (0.79 to 1.02)	0.85 (0.72 to 1.00)	0.90 (0.65 to 1.25)	0.15	
**Milk chocolate**¶						
Age (years):						
<70	1	0.96 (0.89 to 1.04)	0.98 (0.89 to 1.07)	0.88 (0.72 to 1.06)	0.35	0.71
≥70	1	1.01 (0.87 to 1.17)	1.05 (0.88 to 1.26)	1.28 (0.88 to 1.86)	0.22	
Sex:						
Male	1	1.05 (0.85 to 1.29)	0.95 (0.74 to 1.22)	1.00 (0.61 to 1.63)	0.69	0.63
Female	1	0.96 (0.89 to 1.03)	1.00 (0.91 to 1.09)	0.94 (0.78 to 1.13)	0.86	
BMI:						
<30	1	1.00 (0.89 to 1.11)	1.00 (0.87 to 1.14)	0.99 (0.75 to 1.31)	0.94	0.88
≥30	1	0.97 (0.89 to 1.07)	1.01 (0.90 to 1.12)	0.92 (0.74 to 1.16)	0.81	
Physical activity§:						
<Median	1	0.95 (0.87 to 1.04)	1.01 (0.91 to 1.12)	0.98 (0.79 to 1.20)	0.70	0.88
≥Median	1	1.00 (0.89 to 1.12)	0.98 (0.85 to 1.13)	0.87 (0.64 to 1.19)	0.43	
AHEI§:						
<Median	1	1.04 (0.94 to 1.14)	1.06 (0.95 to 1.18)	1.04 (0.85 to 1.27)	0.53	0.23
≥Median	1	0.91 (0.82 to 1.02)	0.94 (0.81 to 1.08)	0.83 (0.57 to 1.23)	0.33	
Family history of diabetes						
No	1	0.98 (0.90 to 1.07)	0.99 (0.90 to 1.10)	0.94 (0.76 to 1.15)	0.67	0.76
Yes	1	0.93 (0.81 to 1.06)	1.01 (0.87 to 1.19)	0.94 (0.68 to 1.30)	0.87	

Data from three cohorts were combined to run the analysis. All models were fully adjusted except for the variable used for stratification. BMI stratified models were further adjusted for baseline BMI as a continuous variable to reduce any residual confounding by baseline BMI. Fully adjusted models adjusted for age, calendar year, ethnicity (white, African American, Asian, and others), smoking status (never, former, current (1–14, 15–24, or ≥25 cigarettes/day), or missing), alcohol intake (g/day: 0, 0.1-4.9, 5.0-14.9, and ≥15.0 in women, 0, 0.1-4.9, 5.0-14.9, 15.0-29.9, and ≥30.0 in men, or missing), family history of diabetes (yes/no), menopausal status and postmenopausal hormone use (premenopausal, postmenopausal (never, former, or current hormone use), or missing, for women), use of oral contraceptives (yes, no, NHSII only), physical activity (<3, 3.0-8.9, 9.0-17.9, 18.0-26.9, ≥27.0 MET-h/week, or missing), baseline BMI (<21.0, 21.0-22.9, 23.0-24.9, 25.0-26.9, 27.0-29.9, 30.0-32.9, 33.0-34.9, ≥35.0, or missing), multivitamin use (yes/no), baseline hypertension, baseline hypercholesterolemia, total energy intake, AHEI (five groups), total chocolate intake before baseline (as cumulative averages between 1980 and 2002 (NHS), 1991-2003 (NHSII), 1986-2002 (HPFS)), and study origin (NHS, NHSII, HPFS).

AHEI=Alternate Healthy Eating Index; BMI=body mass index; NHS=Nurses’ Health Study; NHSII=Nurses’ Health Study II; HPFS=Health Professionals Follow-up Study; MET-h=metabolic equivalent tasks per hour.

* Calculated using Cox proportional hazards models.

† P values for trend were calculated using median levels of chocolate consumption categories as continuous predictor in the model. P values for interaction were obtained by likelihood ratio test.

‡ For total chocolate, baseline years were 1986 for NHS, 1991 for NHSII, and 1986 for HPFS.

§ Stratified levels of physical activity and AHEI were based on median values in each cohort.

¶ For dark and milk chocolate, baseline years were 2006 for NHS, 2007 for NHSII, and 2006 for HPFS.

### Sensitivity analyses

In sensitivity analyses comparing extreme groups, using baseline chocolate consumption level as a non-time varying intake in the model largely weakened the associations between dark chocolate and risk of T2D (hazard ratio 0.90 (95% CI 0.75 to 1.07)), whereas this association was slightly strengthened when modeling dark chocolate consumption based on the most recent update (0.75, 0.63 to 0.90) *(* table 6). Overall results remained unchanged after adjusting for baseline BMI as a continuous variable or time varying categorical BMI instead of baseline categorical BMI *(* table 6 and supplementary table S2). Adjusting for waist circumference appeared to strengthen the association between dark chocolate and risk of T2D, and further attenuated the milk chocolate association towards the null (supplementary table S3). Adjusting for specific foods and drinks high in flavan-3-ols instead of overall dietary quality also did not appear to significantly change the findings. Adjusting for specific foods that predicted T2D slightly attenuated the results toward the null. Further adjustment for epicatechin levels attenuated the associations between dark chocolate and risk of T2D in the highest consumption group (hazard ratio 0.86 (95% CI 0.71 to 1.04)). Findings did not change after further adjustment for other individual flavonoids (supplementary table S4). Adjusting for added sugar slightly attenuated the associations between dark chocolate and risk of T2D (supplementary table S4). Further adjustment for saturated fat intake appeared to strengthen the association for intake of both total chocolate and chocolate subtypes and risk of T2D (total chocolate: 0.88 (0.81 to 0.96), dark chocolate: 0.77 (0.64 to 0.93), milk chocolate: 0.92 (0.78 to 1.10)). In addition, the associations persisted when adjusting for neighborhood socioeconomic status in two female cohorts (NHS and NHSII; supplementary table S5) and when adjusting for educational levels in NHS or specialties of professions in HPFS (supplementary table S6).

**Table 6:** Sensitivity analysis. Values are hazard ratio (95% CI)* unless stated otherwise

Models†	Chocolate consumption levels				P trend‡	Per serving/week
	0 or <1 serving/month	1 serving/month to <1 serving/week	1-4 servings/week	≥5 servings/week		
**Total chocolate§**						
Case/person years	4517/1 298 191	8387/2 094 735	5307/1 263 381	651/1 72 868		
Baseline chocolate	1	1.00 (0.96 to 1.03)	1.00 (0.96 to 1.04)	0.92 (0.85 to 0.99)	0.14	0.99 (0.98 to 1.00)
Most recently updated chocolate	1	1.00 (0.97 to 1.04)	0.99 (0.95 to 1.03)	0.90 (0.84 to 0.96)	0.003	0.99 (0.98 to 1.00)
Adjustment factors:						
Time varying BMI	1	1.01 (0.97 to 1.05)	1.00 (0.96 to 1.04)	0.88 (0.81 to 0.96)	0.01	0.99 (0.97 to 1.00)
Flavan-3-ols-rich food	1	1.04 (1.00 to 1.08)	1.03 (0.99 to 1.07)	0.90 (0.82 to 0.98)	0.05	0.99 (0.98 to 1.00)
Food predicting T2D	1	1.02 (0.98 to 1.06)	1.01 (0.97 to 1.06)	0.91 (0.84 to 1.00)	0.11	0.99 (0.98 to 1.00)
Epicatechin intake	1	1.03 (0.99 to 1.07)	1.02 (0.98 to 1.07)	0.90 (0.83 to 0.99)	0.07	0.99 (0.98 to 1.00)
Saturated fat intake	1	1.03 (0.99 to 1.06)	1.01 (0.96 to 1.05)	0.88 (0.81 to 0.96)	0.01	0.98 (0.97 to 1.00)
**Dark chocolate**¶						
Case/person years	2391/592 412	1473/398 618	776/226 516	131/52 802		
Baseline chocolate	1	0.99 (0.92 to 1.06)	0.91 (0.84 to 0.99)	0.90 (0.75 to 1.07)	0.03	0.98 (0.96 to 1.00)
Most recently updated chocolate	1	0.96 (0.89 to 1.03)	0.93 (0.86 to 1.01)	0.75 (0.63 to 0.90)	0.001	0.97 (0.95 to 0.99)
Adjustment factors:						
Time varying BMI	1	0.96 (0.89 to 1.02)	0.94 (0.86 to 1.02)	0.80 (0.67 to 0.96)	0.01	0.97 (0.95 to 0.99)
Flavan-3-ols rich food	1	0.95 (0.89 to 1.02)	0.92 (0.84 to 1.00)	0.78 (0.65 to 0.93)	0.003	0.97 (0.94 to 0.99)
Food predicting T2D	1	0.95 (0.89 to 1.02)	0.93 (0.85 to 1.01)	0.82 (0.68 to 0.98)	0.01	0.97 (0.95 to 0.99)
Epicatechin intake	1	0.96 (0.90 to 1.03)	0.95 (0.87 to 1.04)	0.86 (0.71 to 1.04)	0.09	0.98 (0.95 to 1.00)
Saturated fat intake	1	0.95 (0.89 to 1.01)	0.91 (0.84 to 1.00)	0.77 (0.64 to 0.93)	0.002	0.96 (0.94 to 0.99)
**Milk chocolate¶**						
Case/person years	1525/476 596	1909/494 537	1168/260 942	169/38 275		
Baseline chocolate	1	0.97 (0.90 to 1.04)	0.99 (0.91 to 1.07)	0.88 (0.74 to 1.04)	0.35	0.99 (0.97 to 1.01)
Most recently updated chocolate	1	0.94 (0.87 to 1.01)	0.98 (0.91 to 1.07)	0.96 (0.81 to 1.14)	0.91	0.99 (0.97 to 1.01)
Adjustment factors:						
Time varying BMI	1	0.97 (0.90 to 1.04)	0.99 (0.91 to 1.08)	0.93 (0.79 to 1.11)	0.70	0.99 (0.97 to 1.01)
Flavan-3-ols rich food	1	0.98 (0.91 to 1.05)	1.00 (0.92 to 1.09)	0.95 (0.80 to 1.13)	0.84	0.99 (0.97 to 1.01)
Food predicting T2D	1	0.97 (0.90 to 1.04)	1.00 (0.92 to 1.09)	0.99 (0.83 to 1.18)	0.82	0.99 (0.97 to 1.02)
Epicatechin intake	1	0.97 (0.90 to 1.04)	0.99 (0.91 to 1.08)	0.95 (0.80 to 1.13)	0.85	0.99 (0.97 to 1.01)
Saturated fat intake	1	0.96 (0.89 to 1.03)	0.98 (0.90 to 1.07)	0.92 (0.78 to 1.10)	0.56	0.98 (0.96 to 1.01)

Data from three cohorts were combined to run the analysis. Fully adjusted models adjusted for age, calendar year, ethnicity (white, African American, Asian, and others), smoking status (never, former, current (1-14, 15-24, or ≥25 cigarettes/day), or missing), alcohol intake (g/day: 0, 0.1-4.9, 5.0-14.9, and ≥15.0 in women, 0, 0.1-4.9, 5.0-14.9, 15.0-29.9, and ≥30.0 in men, or missing), family history of diabetes (yes/no), menopausal status and postmenopausal hormone use (premenopausal, postmenopausal (never, former, or current hormone use), or missing, for women), use of oral contraceptives (yes, no, NHSII only), physical activity (<3, 3.0-8.9, 9.0-17.9, 18.0-26.9, ≥27.0 MET-h/week, or missing), baseline BMI (<21.0, 21.0-22.9, 23.0-24.9, 25.0-26.9, 27.0-29.9, 30.0-32.9, 33.0-34.9, ≥35.0, or missing), multivitamin use (yes/no), baseline hypertension, baseline hypercholesterolemia, total energy intake, alternative health eating index (five groups), total chocolate intake before baseline (as cumulative averages for 1980-2002 (NHS), 1991-2003 (NHSII), 1986-2002 (HPFS)), and study origin (NHS, NHSII, HPFS).

Adjustment of time varying BMI: Comparing with fully adjusted model, instead of adjusting for baseline BMI, adjusted for time varying BMI (<21.0, 21.0-22.9, 23.0-24.9, 25.0-26.9, 27.0-29.9, 30.0-32.9, 33.0-34.9, ≥35.0, or missing).

Adjustment of flavan-3-ols-rich foods: Comparing with fully adjusted model, instead of adjusting for alternative health eating index, adjusted for consumption of food and drinks high in flavan-3-ols, including tea, red wine, apples, pears, blueberries, bananas, and peppers (continuous, servings/day).

Adjustment of food predicting T2D: Comparing with fully adjusted model, instead of adjusting for alternative health eating index, adjusted for consumption of red and processed meat, fruit and vegetables, sugar sweetened beverages, whole grains (fifths).

Adjustment of epicatechin intake: Comparing with fully adjusted model, further adjusting for epicatechin intake (continuous, mg/day).

Adjustment of saturated fat intake: Comparing with fully adjusted model, further adjusting for saturated fat intake (as percentage of total energy intake, five groups).

BMI=body mass index; CI=confidence interval; NHS=Nurses’ Health Study; NHSII=Nurses’ Health Study II; HPFS=Health Professionals Follow-up Study; MET-h=metabolic equivalents task per hour; T2D=type 2 diabetes.

* Calculated using Cox proportional hazards models.

‡ Calculated using median levels of chocolate consumption categories as continuous predictor in the model.

§ For total chocolate, baseline years were 1986 for NHS, 1991 for NHSII, and 1986 for HPFS.

¶ For dark and milk chocolate, baseline years were 2006 for NHS, 2007 for NHSII, and 2006 for HPFS.

† Baseline chocolate: Comparing with fully adjusted model instead of using time dependent intake, use baseline chocolate intake.

Most recently updated chocolate: Comparing with fully adjusted model instead of using cumulatively averaged intake, use most recently updated chocolate intake.

### Chocolate intake and weight change

Table 7 shows the results for bodyweight change analyses by chocolate subtypes. Compared with those who did not change their chocolate intake, increased intake of milk chocolate over four year periods was associated with 0.35 kg (95% CI 0.27 to 0.43) more four year weight gain over time. Increasing dark chocolate intake was not associated with weight change over time (−0.06 kg, −0.13 to 0.02). The association between changes in total chocolate intake with long term weight change was also positive (supplementary table S7). The results across the three cohorts were largely consistent, although the associations for total and milk chocolate were the strongest in NHSII compared with the other two cohorts (supplementary table S8).

**Table 7:** Weight change over four year periods according to four year change in dark and milk chocolate intake

	Increase				Decrease		
	β*	SE (95% CI)	P value†		β*	SE (95% CI)	P value†
**Dark chocolate‡**							
Model 1	0	0.04 (−0.07 to 0.08)	0.93		−0.07	0.03 (−0.14 to −0.00)	0.04
Model 2	−0.06	0.04 (−0.13 to 0.02)	0.14		−0.08	0.04 (−0.17 to −0.00)	0.04
**Milk chocolate‡**							
Model 1	0.44	0.04 (−0.38 to −0.25)	<0.001		−0.32	0.03 (−0.38 to −0.25)	<0.001
Model 2	0.35	0.04 (0.27 to 0.43)	<0.001		−0.40	0.04 (−0.47 to −0.32)	<0.001

Multivariable generalized linear regression models (with independent correlation matrix and robust variance) were used.

Model 1: Adjusted for age.

Model 2: Additionally adjusted for ethnicity (white, African American, Asian, and others), family history of diabetes, baseline hypertension, hypercholesterolemia, baseline and change in total energy intake, change in smoking status (stayed never smoker, stayed former smoker, stayed current smoker, changed from former to current smoker, changed from never to current smoker, and changed from current to former smoker), baseline and change in physical activity (MET-h/week), change in alcohol consumption (continuous, g/day), postmenopausal hormone use (women only; premenopausal, never, former, current, or missing), oral contraceptive use (NHSII only; yes, no), changes in alternative healthy eating index (AHEI, five groups), and study origin (NHS, NHSII, HPFS).

AHEI=Alternate Healthy Eating Index; CI=confidence interval; SE=standard error; NHS=Nurses’ Health Study; NHSII=Nurses’ Health Study II; HPFS=Health Professionals Follow-up Study; MET-h=metabolic equivalent tasks per hour; SE=standard error.

* Interpreted as four year weight change in kilograms (95% CI) comparing participants with increased or decreased dark or milk chocolate intake to those with no change in dark or milk chocolate intake over four year periods. Data from three cohorts were combined to run the analysis.

† P values obtained using Wald test.

‡ For dark and milk chocolate, follow-up periods were 2006-10 for NHS, 2007-15 for NHSII, and 2006-18 for HPFS.

In the stratified weight change analyses, we identified baseline BMI as a significant modifier for the associations between four year changes in dark or milk chocolate intake and four year weight change (table 8; P values for interaction <0.001). Increased intake of milk chocolate was associated with more increase in bodyweight in those with obesity at baseline (BMI ≥30) compared with those with a BMI within normal range (<25). Specifically, increased consumption of milk chocolate compared with no change in milk chocolate intake was associated with an average weight gain of 0.68 kg (0.42 to 0.95) more in people with obesity, whereas weight gain in those with normal BMI was only 0.33 kg (0.24 to 0.42) more over four year intervals. Increased dark chocolate intake was not associated with long term weight change in any BMI subgroups. The associations for total chocolate were also stronger in the group with a higher BMI (supplementary table S7). In the primary analyses for diabetes outcome, however, adjusting for time varying BMI did not explain the associations for any chocolate intake and risk of T2D.

**Table 8:** Weight change over four year periods according to four year change in chocolate subtype intake, stratified by baseline BMI

	No of participants	Increase				Decrease			P for interaction‡
		β*	SE (95% CI)	P value†		β*	SE (95% CI)	P value†	
**Dark chocolate**									
BMI:									
<25	39 108	−0.06	0.04 (−0.14 to 0.01)	0.10		0.06	0.04 (−0.02 to 0.15)	0.14	<0.001
25-30	29 007	−0.13	0.06 (−0.25 to −0.01)	0.03		−0.10	0.06 (−0.22 to 0.02)	0.11	
≥30	17 690	−0.07	0.14 (−0.34 to 0.19)	0.60		−0.45	0.14 (−0.72 to −0.17)	0.001	
**Milk chocolate**									
BMI:									
<25	39 108	0.33	0.04 (0.24 to 0.42)	<0.001		−0.05	0.04 (−0.12 to 0.02)	<0.001	<0.001
25-30	29 007	0.27	0.06 (0.14 to 0.39)	<0.001		−0.37	0.06 (−0.49 to −0.26)	<0.001	
≥30	17 690	0.68	0.14 (0.42 to 0.95)	<0.001		−0.85	0.13 (−1.10 to −0.59)	<0.001	

Multivariable generalized linear regression models (with independent correlation matrix and robust variance) were used.

All models adjusted for age, ethnicity (white, African American, Asian, and others), baseline BMI (continuous), family history of diabetes, baseline hypertension, hypercholesterolemia, baseline and change in total energy intake, change in smoking status (stayed never smoker, stayed former smoker, stayed current smoker, changed from former to current smoker, changed from never to current smoker, and changed from current to former smoker), baseline and change in physical activity (MET-h/week), change in alcohol consumption (continuous, g/day), postmenopausal hormone use (women only; premenopausal, never, former, current, or missing), oral contraceptive use (NHSII only; yes, no), changes in AHEI (five groups), and study origin (NHS, NHSII, HPFS).

AHEI=Alternate Healthy Eating Index; BMI=body mass index; CI=confidence interval; NHS=Nurses’ Health Study; NHSII=Nurses’ Health Study II; HPFS=Health Professionals Follow-up Study; MET-h=metabolic equivalent tasks per hour; SE=standard error.

* Interpreted as four year weight change in kilograms (95% CI) comparing participants with increased or decreased total chocolate intake to those with no change in total chocolate intake over four year periods. Data from three cohorts were combined to run this analysis.

† Calculated using Wald test.

‡ Calculated using generalized score tests.

## Discussion

The findings of the present study showed that higher consumption of dark, but not milk, chocolate was associated with a lower risk of T2D. Spline regression analysis showed a linear dose-response association between intake of dark chocolate and risk of T2D (P for linearity=0.003). These findings were independent of established and potential risk factors for diabetes and were robust in multiple sensitivity analyses, although statistically significant heterogeneity was also noted among cohorts. Stratified analyses indicated that the association of dark chocolate intake was more apparent among younger individuals. Epicatechin intake may partially account for the inverse associations of dark chocolate. Similar differential associations between milk and dark chocolate intake were also observed for change in bodyweight. Milk chocolate consumption was statistically significantly associated with more weight gain but increased dark chocolate intake was not associated with weight gain. In fact, time varying BMI did not explain the associations for dark chocolate and risk of T2D in our data.

### Comparison with other studies

Our study’s finding that intake of total chocolate was statistically significantly associated with lower risk of diabetes was in line with previously published studies. The Physicians’ Health Study reported that consuming ≥2 servings/week of total chocolate was associated with a 17% (95% CI 1% to 31%) lower risk of T2D among 18 235 participants during a median follow-up of 9.2 years.^16^ In the Multiethnic Cohort Study, consuming chocolate ≥4 times/week was associated with a 19% (95% CI 9% to 28%) lower risk of T2D compared with a lower frequency of consumption (<once/month).^41^ In the Maine-Syracuse Longitudinal Study, individuals who never or rarely ate chocolate had a 91% (95% CI 3% to 255%) higher risk of T2D than those who consumed chocolate more than once per week.^42^

Dark and milk chocolate could have a differential effect on T2D; however, evidence for associations pertinent to intake of chocolate subtypes is sparse. The National Health and Nutrition Examination Survey (2007-08 and 2013-14) found that 11.1% of US adults consumed chocolate on a regular basis, whereas only 1.4% reported consuming dark chocolate (≥45% cocoa content).^43^ Major subtypes of chocolate (dark, milk, and white) differ mainly in their cocoa and sugar content and presence of milk.^12^ Among the three subtypes of chocolate products, dark chocolate has the highest cocoa content (50-80% cacao) and is the richest chocolate in flavan-3-ols (mean 3.65 mg/g).^44^ Milk chocolate comprises less than one fifth of the flavan-3-ols (mean 0.69 mg/g) in dark chocolate, has a lower cocoa content (~35%), and has a higher sugar content.^44^ White chocolate has the highest sugar content, and, since it does not contain cocoa, has no polyphenols. Flavan-3-ol content of chocolate is highly variable depending on the type of processing used. The current analysis investigated the association between chocolate subtypes and risk of T2D, and we found evidence of a linear, inverse association of dark chocolate intake with risk of T2D. Our findings are largely in line with randomized controlled trials that examined dark chocolate or cocoa in relation to T2D or cardiometabolic risk factors. For example, a 15 day randomized controlled trial in glucose intolerant, hypertensive participants found that daily consumption of 100 g high polyphenol dark chocolate led to significant reductions in blood pressure and improvements in insulin sensitivity, compared with a placebo group consuming 90 g white chocolate.^45^ Studies examining the consumption of high versus low flavanol cocoa products also showed health benefits in improving insulin resistance among individuals with overweight and obesity,^46^ increasing high density lipoprotein cholesterol levels, and lowering blood pressure among patients with diabetes.^47^ ^48^ However, the recent large scale randomized controlled trial, COSMOS (cocoa supplement and multivitamin outcomes study), which administered 500 mg cocoa flavanols supplements per day (including 80 mg epicatechin) among 21 442 US men and women aged, on average, 72.0 years, yielded somewhat unexpected findings after a median of 3.5 years of follow-up. Flavanol supplementation significantly lowered the risk of death due to cardiovascular disease compared with placebo (hazard ratio 0.73, 95% CI 0.54 to 0.98), but no effects were found on risk of T2D (1.04, 0.91 to 1.20).^10^ ^49^ Reasons for the discrepancy between our observations and the COSMOS findings are unknown. Meanwhile, the inverse associations between dark chocolate intake and risk of T2D in our cohorts were primarily observed among younger individuals (<70 years old) (table 5). Further studies are warranted to elucidate potential age specific effects of cocoa or flavanol intake.

Furthermore, our results suggest that increasing total chocolate intake was associated with more gain in bodyweight, but this was likely mainly driven by milk chocolate. These findings were consistent with some previously published longitudinal studies on total chocolate. In the Atherosclerosis Risk in Communities cohort, every 1 oz/day intake of total chocolate was associated with 0.19 (95% CI 0.04 to 0.15) more change in BMI over six years of the study period.^14^ In the Women’s Health Initiative, each 1 oz/day intake of chocolate candy and candy bar was associated with 0.92 kg (95% CI 0.80 to 1.05) more three year weight gain.^50^ Another intriguing observation in our study was the heterogeneity across the three cohorts, with more statistically significant results observed in men than in elderly postmenopausal women, consistent with previously published sex stratified results.^51^ Oba and colleagues reported similar findings to ours among Japanese people; that consumption of chocolate once or more per week was associated with a 35% (95% CI 3% to 57%) lower risk of T2D in men but no significant effect in women (0.73, 0.48 to 1.13), compared with those who never ate chocolate.^51^ The reasons underlying these potential gender differences are unknown, although sex hormones might play a role in modifying the association between chocolate consumption and risk of T2D. These findings might also be ascribed to milk chocolate as the primary type of chocolate consumed by study participants, yet neither of these longitudinal studies differentiated between subtypes of chocolate.^50^ In contrast with total or milk chocolate intake, our study showed that increasing dark chocolate intake was not associated with more weight gain over ≥20 years of follow-up. This finding was consistent with evidence from previous short term (≤8 weeks) randomized controlled trials, which showed no significant weight changes after regular intake of dark chocolate.^45^ ^52^

Dark chocolate, with a higher cocoa content than milk chocolate, may lower the risk of T2D through various mechanisms.^53^ It has been suggested that bioactive compounds in cocoa, such as flavan-3-ols and their monomeric form, epicatechin, mitigate risk of T2D by improving insulin sensitivity,^11^ ^54^ protecting pancreatic β cells from oxidative stress,^55^ lowering pro-inflammatory cytokines such as tumor necrosis factor-α and IL-6,^56^ and improving endothelial function by stimulating the production of nitric oxide, a vasodilator, which may lead to improved glucose metabolism and reduced risk of T2D.^57^ ^58^ ^59^ Milk and white chocolate intake might not lead to the same metabolic health benefits owing to their higher added sugar content—an established dietary risk factor for cardiometabolic diseases.^60^ ^61^ In contrast, although dark chocolate contains similar levels of energy and saturated fat to milk chocolate (mean percentage of fat in commercially available chocolate (dark: 34.7%; milk: 32.6%^44^), the rich polyphenols in dark chocolate might offset the effects of saturated fat and sugar on weight gain and the risk of other cardiometabolic diseases.^62^

### Strengths and limitations of this study

A major strength of our study is that we differentiated chocolate intake by subtypes and examined their associations with risk of T2D and weight change among healthy individuals over long follow-up periods. However, our study also has several limitations. First, we cannot entirely rule out the role of confounding in our observed associations. We controlled for multiple lifestyle and dietary covariates that might confound the associations of interest, although residual or unmeasured confounding, or both, may still exist owing to the observational nature of the analysis. Second, the relatively limited number of people with T2D in the higher chocolate consumption groups may have led to reduced statistical power for detecting modest associations between dark chocolate consumption and risk of T2D. Third, most of our study population for chocolate subtype analyses were non-Hispanic white adults older than 50 years at baseline, which, together with their professions, may limit the generalizability of our findings to other populations with different socioeconomic or personal characteristics. Nevertheless, the observed associations persisted when further adjusting for neighborhood z scores for socioeconomic status, educational levels, and specialty of professions. Fourth, chocolate consumption was relatively low in our study population, compared with the national average in the US Department of Agriculture Nationwide Food Consumption Survey 1987-88 of about three servings/week.^63^ This may have hindered our ability to assess the dose-response association at higher intake. Lastly, food frequency questionnaires are subject to measurement errors, although the prospective design may make this error non-differential, and, as such, the associations may be more likely to be biased toward the null.

### Conclusion and policy implications

Intake of dark chocolate instead of milk chocolate may be associated with a lower risk of T2D. Increased consumption of milk chocolate but not dark chocolate, however, was associated with increased weight gain. Further research, especially randomized controlled trials among middle aged participants and of longer duration, is needed to confirm these findings.

**What is already known on this topic**

Chocolate contains high levels of flavanols, which promote cardiometabolic health and reduce the risk of type 2 diabetes (T2D), as shown in randomized controlled trials
The associations between chocolate consumption and risk of T2D remain controversial owing to inconsistent findings in observational studies
Most previous studies did not differentiate between chocolate subtypes (dark, milk), which differ in their cocoa content and proportions of other ingredients such as sugar and milk, and may have differential associations with risk of T2D

**What this study adds**

Consumption of ≥5 servings/week of dark chocolate compared with rare consumption was statistically significantly associated with lower risk of T2D
The association for milk chocolate was, however, null
Increased consumption of milk chocolate but not dark chocolate was associated with increased weight gain

## Data Availability

No additional data available.

## Web extra

Extra material supplied by authors

## Notes

Contributors: BL, GZ, and QS designed the study. JEM, FBH, and QS collected the data. BL and GZ conducted the data analyses. BL prepared the first draft of the manuscript. All authors contributed to the interpretation of the results and critical revision of the manuscript for important intellectual content and approved the final version of the manuscript. QS is the guarantor. The corresponding author attests that all listed authors meet authorship criteria and that no others meeting the criteria have been omitted.

Transparency: The first author (BL) affirms that the manuscript is an honest, accurate, and transparent account of the study being reported; that no important aspects of the study have been omitted; and that any discrepancies from the study as planned have been explained.

Dissemination to participants and related patient and public communities: There are no plans to disseminate the results of the research to study participants or the relevant patient community. The participants are updated on the study outcomes and developments through the study websites (www.nurseshealthstudy.org and www.hsph.harvard.edu/hpfs) and newsletters.

Provenance and peer review: Not commissioned; externally peer reviewed.
