# Supplementary material for: Chocolate intake and risk of type 2 diabetes: prospective cohort studies
Source: BMJ. 2024 Dec 4;387:e078386. doi: 10.1136/bmj-2023-078386 (PMC11616007; doi:10.1136/bmj-2023-078386)
Supplement: Supplementary file 1 — Supplementary information: Additional tables S1-S8 and figures S1 and S2 [file liub078386.ww.pdf]

Table S1. Age-standardized baseline characteristics according to total chocolate consumption at baseline.*

	NHS				NHSII				HPFS			
Frequency	**Never or <1 serving/ month**	**1 serving/month to <1 serving/week**	**1-4 servings/ week**	**≥5 servings/ week**	**Never or <1 serving/ month**	**1 serving/month to <1 serving/week**	**1-4 servings/ week**	**≥5 servings/ week**	**Never or <1 serving/ month**	**1 serving/month to <1 serving/week**	**1-4 servings/ week**	**≥5 servings/ week**
**Total chocolate[1]**												
n	28554	21337	12198	1709	24712	32309	27203	4159	16246	12584	9687	1510
Total chocolate[1], serving/week	0 (0)	0.5 (0)	1.7 (1)	7.9 (5.2)	0 (0)	0.5 (0)	1.7 (1.0)	7.1 (3.2)	0 (0)	0.5 (0)	1.7 (1.0)	7.9 (4.8)
Age, years	52.9 (7.1)	51.8 (7.1)	51.4 (7.1)	52.3 (7.3)	36.6 (4.6)	36 (4.7)	35.8 (4.7)	35.8 (4.6)	53.9 (9.5)	52.7 (9.5)	52.2 (9.6)	53.0 (9.6)
White, %(n)	97.6 (27862)	97.9 (20887)	98.4 (12000)	98.7 (1686)	94.8 (23429)	95.4 (30816)	96.7 (26313)	97.0 (4036)	94.5 (15350)	95.3 (11992)	96.1 (9308)	95.8 (1446)
Current smoking, %(n)	20.3 (5788)	20.2 (4320)	22.5 (2740)	28.5 (487)	12.2 (3024)	11.9 (3849)	12.5 (3390)	12.1 (505)	9.1 (1482)	9.3 (1170)	10.2 (986)	11.6 (175)
Alcohol intake (g/day)	7.4 (12.0)	5.7 (9.9)	4.8 (8.5)	4.6 (8.3)	3.5 (7.0)	3.2 (6.0)	2.9 (5.5)	2.4 (4.9)	13.0 (16.8)	10.9 (14.6)	10.1 (13.9)	9.2 (14.0)
Median (SD) physical activity (MET-h/week)	15.0 (21.0)	13.8 (19.9)	12.9 (19.0)	13.8 (26.9)	22.9 (29.6)	20.6 (26.8)	19.7 (25.8)	20.3 (29.1)	22.0 (25.5)	20.2 (24.3)	20.5 (25.0)	20.5 (27.0)
BMI	24.8 (4.5)	25.4 (4.7)	25.5 (4.9)	25.2 (4.9)	23.8 (4.6)	24.5 (5.1)	25 (5.7)	25.2 (6.1)	24.6 (4.9)	25.1 (4.8)	25.0 (4.9)	25.0 (4.5)
Family history of diabetes, %(n)	25.5 (7272)	26.5 (5658)	25.8 (3151)	25.9 (443)	15.8 (3901)	16.0 (5183)	16.0 (4343)	16.2 (675)	18.3 (2972)	19.0 (2394)	17.9 (1730)	19.4 (293)
Multivitamin use, %(n)	43.6 (12457)	41.9 (8950)	40.8 (4974)	40.9 (699)	45.6 (11257)	43.5 (14063)	42.6 (11577)	42.3 (1760)	44.4 (7212)	40.1 (5051)	40.2 (3899)	39.4 (595)
Use of any postmenopausal hormone, %(n)	27.4 (7832)	26.8 (5719)	26.7 (3255)	25.9 (442)	3.0 (742)	3.0 (977)	3.1 (850)	3.0 (126)	/	/	/	/
Use of oral contraceptives, %(n)	/	/	/	/	15.7 (3885)	15.1 (4892)	15.4 (4198)	14.6 (605)	/	/	/	/
Baseline hypertension, %(n)	24.1 (6874)	22.7 (4847)	22.3 (2722)	21.1 (361)	5.7 (1419)	5.8 (1872)	6.5 (1756)	6.9 (286)	19.3 (3140)	19.1 (2410)	19.2 (1863)	18.7 (282)
Baseline hypocholesterolemia, %(n)	12.0 (3417)	11.2 (2390)	11.3 (1375)	11.1 (189)	13.2 (3250)	14.1 (4540)	15.2 (4122)	16.3 (678)	10.6 (1720)	10.3 (1303)	10.1 (980)	8.7 (132)
Total energy intake, kcal/day	1645 (490)	1799 (510)	1947 (539)	2197(569)	1635 (510)	1753 (520)	1910 (552)	2138 (580)	1857 (578)	1989 (595)	2168 (632)	2481 (676)
Alternative healthy eating index	53.7 (11.7)	51.6 (10.9)	50.6 (10.9)	49.7 (11.5)	50.7 (11.2)	47.9 (10.4)	45.8 (10.2)	44.1 (10.3)	54.4 (11.8)	52.2 (11.1)	50.6 (10.9)	49.0 (11.3)

Red or processed meats, serving/day	0.9 (0.6)	1.1 (0.7)	1.2 (0.7)	1.2 (0.7)	0.8 (0.6)	0.9 (0.6)	1.1 (0.7)	1.1 (0.7)	1.0 (0.8)	1.2 (0.8)	1.3 (0.9)	1.4 (0.9)
Fruit and vegetables, serving/day	5.4 (2.9)	5.3 (2.7)	5.2 (2.6)	4.9 (2.6)	4.4 (2.7)	4.1 (2.3)	4.0 (2.2)	3.8 (2.2)	5.1 (3.0)	4.8 (2.7)	4.7 (2.5)	4.5 (2.5)
Whole grain, serving/day	1.2 (1.1)	1.1 (1.1)	1.1 (1.0)	1 (1.1)	1.4 (1.2)	1.4 (1.1)	1.3 (1.1)	1.3 (1.1)	1.5 (1.5)	1.4 (1.3)	1.3 (1.3)	1.3 (1.3)
Sugar sweetened beverage, serving/day	0.2 (0.5)	0.2 (0.5)	0.3 (0.6)	0.4 (0.7)	0.4 (0.8)	0.5 (0.8)	0.6 (0.9)	0.7 (1.1)	0.3 (0.5)	0.4 (0.6)	0.5 (0.7)	0.7 (0.9)
Tea, serving/day	0.6 (1.0)	0.6 (1.0)	0.6 (1.0)	0.7 (1.2)	0.7 (1.1)	0.7 (1.1)	0.7 (1.1)	0.7 (1.1)	0.4 (0.9)	0.4 (0.9)	0.4 (0.8)	0.4 (0.9)
Coffee, serving/day	2.4 (1.7)	2.4 (1.8)	2.5 (1.8)	2.4 (1.9)	1.6 (1.7)	1.6 (1.7)	1.5 (1.7)	1.4 (1.7)	1.9 (1.8)	1.9 (1.8)	1.9 (1.8)	2.1 (1.9)
Sweets and desserts, serving/day	0.8 (0.9)	1.1 (1.0)	1.7 (1.2)	3.1 (1.8)	0.8 (0.8)	1.1 (0.9)	1.6 (1.1)	2.8 (1.5)	0.7 (1.0)	1.1 (1.1)	1.6 (1.3)	3.1 (1.9)
Saturated fat, % energy	11.2 (2.7)	11.8 (2.4)	12.3 (2.3)	13.8 (2.5)	10.4 (2.5)	11.1 (2.3)	11.7 (2.2)	13.4 (2.4)	10.3 (2.9)	11.1 (2.5)	11.8 (2.4)	13.2 (2.5)
Added sugar, g/day	32.2 (18.2)	36.5 (16.6)	40.7 (16.3)	48.8 (18.2)	49.7 (30.4)	53.6 (28.7)	58.1 (28)	67.4 (28.7)	41.4 (25.4)	48.6 (23.6)	55.6 (24.5)	67.1 (24.8)
Cereal fiber, g/day	4.6 (3.4)	4.4 (2.9)	4.2 (2.6)	3.7 (2.3)	6.1 (3.6)	5.6 (3.0)	5.3 (2.6)	4.8 (2.5)	6.3 (4.5)	5.8 (3.5)	5.5 (3.5)	4.8 (2.7)
Epicatechin, mg/day	9.1 (7.1)	8.6 (6.1)	8.2 (5.6)	8.6 (5.6)	9.9 (8.3)	9.3 (7.3)	8.9 (6.6)	9 (6.0)	9.4 (7.4)	8.8 (6.5)	8.4 (5.8)	8.9 (5.6)
Total Flavonoids, mg/day	355.4 (337.1)	337.5 (302.5)	318.2 (280.4)	327 (283.4)	399.3 (411.4)	381.7 (375.4)	361.4 (340.2)	346.9 (304.2)	338.6 (302.9)	321.4 (276.1)	302.7 (249.0)	309.7 (240.1)

Abbreviations: MET: metabolic equivalent tasks; BMI: body mass index; NHS: Nurses' Health Study; NHSII: Nurses' Health Study II; HPFS: Health Professionals Follow-up Study.
* Values are means (SD) or percentages (n) and are standardized to the age distribution of the study population.
[1]For total chocolate, baseline years were 1986 for NHS, 1991 for NHSII, and 1986 for HPFS.
[2]Median (SD).

Table S2. Sensitivity analysis further adjusting for baseline BMI as a continuous variable.

	Chocolate subtypes consumption levels					
	Never or < 1 serving/month	1 serving/month to <1 serving/week	1-4 servings/week	≥5 servings/week	P trend‡	per serving/week
Dark chocolate	1	0.97 (0.90 to 1.04)	0.92 (0.85 to 1.01)	0.78 (0.65 to 0.94)	0.003	0.97 (0.94 to 0.99)
Milk chocolate	1	1.00 (0.93 to 1.07)	1.01 (0.93 to 1.10)	0.94 (0.79 to 1.12)	0.72	0.98 (0.96 to 1.01)

Data from three cohorts were combined to run this analysis. Values are hazards ratios (95% confidence interval) calculated in Cox proportional hazards models.
‡ P trend values were calculated using median levels of chocolate consumption categories as the continuous predictor in the model.

All models adjusted for age, calendar year, ethnicity (White, African American, Asian, and others), smoking status (never, former, current (1–14, 15–24, or ≥25 cigarettes/day), or missing), alcohol intake (gram/day: 0, 0.1-4.9, 5.0-14.9, and ≥15.0 in women, 0, 0.1-4.9, 5.0-14.9, 15.0-29.9, and ≥30.0 in men, or missing), family history of diabetes (yes/no), menopausal status and post-menopausal hormone use (pre-menopause, post-menopause (never, former, or current hormone use), or missing, for women), use of oral contraceptives (yes, no, NHSII only), physical activity (<3, 3.0-8.9, 9.0-17.9, 18.0-26.9, ≥27.0 metabolic equivalent of task hours/week, or missing), body mass index (continuous), multivitamin use (yes/no), baseline hypertension, baseline hypercholesterolemia, total energy intake, alternative health eating index (quintile groups), total chocolate intake prior to baseline (as cumulative averages between 1980-2002 (NHS), 1991-2003 (NHSII), 1986-2002 (HPFS)), and study origin (NHS, NHSII, HPFS).

Table S3. Sensitivity analysis further adjusting for waist circumference.

	Chocolate subtypes consumption levels					
	**Never or < 1 serving/month**	**1 serving/month to <1 serving/week**	**1-4 servings/week**	**≥5 servings/week**	**P trend‡**	**per serving/week**
Dark chocolate	1	0.98 (0.91 to 1.05)	0.89 (0.81 to 0.98)	0.72 (0.59 to 0.88)	0.0002	0.95 (0.93 to 0.98)
Milk chocolate	1	1.02 (0.95 to 1.11)	1.01 (0.92 to 1.11)	0.97 (0.80 to 1.17)	0.78	0.99 (0.96 to 1.01)

Data from three cohorts were combined to run this analysis. Values are hazards ratios (95% confidence interval) calculated in Cox proportional hazards models.
‡ P trend values were calculated using median levels of chocolate consumption categories as the continuous predictor in the model.

All models adjusted for age, calendar year, ethnicity (White, African American, Asian, and others), smoking status (never, former, current (1–14, 15–24, or ≥25 cigarettes/day), or missing), alcohol intake (gram/day: 0, 0.1-4.9, 5.0-14.9, and ≥15.0 in women, 0, 0.1-4.9, 5.0-14.9, 15.0-29.9, and ≥30.0 in men, or missing), family history of diabetes (yes/no), menopausal status and post-menopausal hormone use (pre-menopause, post-menopause (never, former, or current hormone use), or missing, for women), use of oral contraceptives (yes, no, NHSII only), physical activity (<3, 3.0-8.9, 9.0-17.9, 18.0-26.9, ≥27.0 metabolic equivalent of task hours/week, or missing), waist circumference (continuous), multivitamin use (yes/no), baseline hypertension, baseline hypercholesterolemia, total energy intake, alternative health eating index (quintile groups), total chocolate intake prior to baseline (as cumulative averages between 1980-2002 (NHS), 1991-2003 (NHSII), 1986-2002 (HPFS)), and study origin (NHS, NHSII, HPFS).

Table S4. Sensitivity analysis further adjusting for added sugar and individual flavonoids.

	Chocolate consumption levels					
Dark chocolate	**Never or <1 serving/month**	**1 serving/month to <1 serving/week**	**1-4 servings/week**	**≥5 servings/week**	**P trend‡**	**Per serving/week**
Additionally adjust for added sugar	1	0.97 (0.90 to 1.03)	0.95 (0.87 to 1.04)	0.83 (0.69 to 1.00)	0.048	0.98 (0.95 to 1.00)
Additionally adjust for individual flavonoids						
Flavonols	1	0.95 (0.89 to 1.02)	0.93 (0.85 to 1.01)	0.79 (0.66 to 0.95)	0.006	0.97 (0.95 to 0.99)
Flavones	1	0.95 (0.89 to 1.02)	0.93 (0.85 to 1.01)	0.79 (0.66 to 0.95)	0.006	0.97 (0.95 to 0.99)
Flavanones	1	0.95 (0.89 to 1.02)	0.93 (0.85 to 1.01)	0.79 (0.66 to 0.95)	0.005	0.97 (0.95 to 0.99)
Flavan-3-ols	1	0.95 (0.89 to 1.02)	0.92 (0.85 to 1.01)	0.79 (0.66 to 0.95)	0.006	0.97 (0.94 to 0.99)
Catechin	1	0.96 (0.89 to 1.03)	0.93 (0.86 to 1.02)	0.80 (0.67 to 0.97)	0.01	0.97 (0.95 to 0.99)
Epicatechin	1	0.96 (0.89 to 1.02)	0.94 (0.86 to 1.03)	0.85 (0.70 to 1.04)	0.07	0.98 (0.95 to 1.00)
Gallocatechin	1	0.95 (0.89 to 1.02)	0.93 (0.85 to 1.01)	0.79 (0.66 to 0.95)	0.01	0.97 (0.95 to 0.99)
Epigallocatechin	1	0.95 (0.89 to 1.02)	0.93 (0.85 to 1.01)	0.79 (0.66 to 0.95)	0.01	0.97 (0.95 to 0.99)
Epicatechin 3-gallate	1	0.95 (0.89 to 1.02)	0.93 (0.85 to 1.01)	0.79 (0.66 to 0.95)	0.01	0.97 (0.95 to 0.99)
Epigallocatechin 3-gallate	1	0.95 (0.89 to 1.02)	0.93 (0.85 to 1.01)	0.79 (0.66 to 0.95)	0.01	0.97 (0.95 to 0.99)
Total theaflavin and polymers proanthocyanidin	1	0.95 (0.89 to 1.02)	0.93 (0.85 to 1.01)	0.79 (0.66 to 0.95)	0.007	0.97 (0.95 to 0.99)
Total Flavonoids	1	0.95 (0.89 to 1.02)	0.93 (0.85 to 1.01)	0.79 (0.66 to 0.95)	0.007	0.97 (0.95 to 0.99)

	Chocolate consumption levels					
Milk chocolate	**Never or <1 serving/month**	**1 serving/month to <1 serving/week**	**1-4 servings/week**	**≥5 servings/week**	**P trend‡**	**Per serving/week**
Additionally adjust for added sugar	1	0.99 (0.92 to 1.07)	1.04 (0.95 to 1.13)	1.01 (0.85 to 1.20)	0.46	1.00 (0.98 to 1.02)
Additionally adjust for nutrients						
Flavonols	1	0.97 (0.90 to 1.04)	1.00 (0.92 to 1.08)	0.95 (0.80 to 1.13)	0.85	0.99 (0.97 to 1.01)
Flavones	1	0.97 (0.90 to 1.04)	1.00 (0.92 to 1.08)	0.95 (0.80 to 1.13)	0.85	0.99 (0.97 to 1.01)
Flavanones	1	0.97 (0.90 to 1.04)	0.99 (0.91 to 1.07)	0.93 (0.79 to 1.11)	0.65	0.99 (0.97 to 1.01)
Flavan-3-ols	1	0.97 (0.90 to 1.04)	0.99 (0.91 to 1.08)	0.94 (0.79 to 1.12)	0.71	0.99 (0.97 to 1.01)
Catechin	1	0.97 (0.90 to 1.04)	0.99 (0.91 to 1.08)	0.94 (0.79 to 1.12)	0.72	0.99 (0.97 to 1.01)
Epicatechin	1	0.96 (0.90 to 1.03)	0.99 (0.91 to 1.07)	0.95 (0.80 to 1.13)	0.77	0.99 (0.97 to 1.01)
Gallocatechin	1	0.97 (0.90 to 1.04)	0.99 (0.91 to 1.08)	0.94 (0.79 to 1.12)	0.75	0.99 (0.97 to 1.01)
Epigallocatechin	1	0.97 (0.90 to 1.04)	0.99 (0.91 to 1.08)	0.94 (0.79 to 1.11)	0.7	0.99 (0.97 to 1.01)
Epicatechin 3-gallate	1	0.97 (0.90 to 1.04)	0.99 (0.91 to 1.08)	0.94 (0.79 to 1.12)	0.72	0.99 (0.97 to 1.01)
Epigallocatechin 3-gallate	1	0.97 (0.90 to 1.04)	0.99 (0.91 to 1.08)	0.94 (0.79 to 1.12)	0.72	0.99 (0.97 to 1.01)

Total theaflavin and polymers proanthocyanidin	1	0.97 (0.90 to 1.04)	0.99 (0.91 to 1.07)	0.94 (0.79 to 1.11)	0.67	0.99 (0.97 to 1.01)
Total Flavonoids	1	0.97 (0.90 to 1.04)	0.99 (0.91 to 1.07)	0.94 (0.79 to 1.12)	0.70	0.99 (0.97 to 1.01)

Data from three cohorts were combined to run this analysis. Values are hazards ratios (95% confidence interval) calculated in Cox proportional hazards models.
‡ P trend values were calculated using median levels of chocolate consumption categories as the continuous predictor in the model.

All models adjusted for age, calendar year, ethnicity (White, African American, Asian, and others), smoking status (never, former, current (1–14, 15–24, or ≥25 cigarettes/day), or missing), alcohol intake (gram/day: 0, 0.1-4.9, 5.0-14.9, and ≥15.0 in women, 0, 0.1-4.9, 5.0-14.9, 15.0-29.9, and ≥30.0 in men, or missing), family history of diabetes (yes/no), menopausal status and post-menopausal hormone use (pre-menopause, post-menopause (never, former, or current hormone use), or missing, for women), use of oral contraceptives (yes, no, NHSII only), physical activity (<3, 3.0-8.9, 9.0-17.9, 18.0-26.9, ≥27.0 metabolic equivalent of task hours/week, or missing), baseline body mass index (BMI) (<21.0, 21.0-22.9, 23.0-24.9, 25.0-26.9, 27.0-29.9, 30.0-32.9, 33.0-34.9, ≥35.0 kg/m^2, or missing), multivitamin use (yes/no), baseline hypertension, baseline hypercholesterolemia, total energy intake, alternative health eating index (quintile groups), and total chocolate intake prior to baseline (as cumulative averages between 1980-2002 (NHS), 1991-2003 (NHSII), 1986-2002 (HPFS)).

Table S5. Sensitivity analysis adjusting for Neighborhood Social Economic Status in NHS and NHSII

		Chocolate subtypes consumption levels					
		Never or < 1 serving/month	1 serving/month to <1 serving/week	1-4 servings/week	≥5 servings/week	P trend‡	per serving/week
Dark chocolate							
**NHS**	Multivariable adjusted	1	0.92 (0.80 to 1.06)	0.99 (0.83 to 1.18)	0.99 (0.68 to 1.43)	0.96	0.99 (0.95 to 1.04)
**NHSII**	Multivariable adjusted	1	0.95 (0.87 to 1.03)	0.91 (0.82 to 1.02)	0.80 (0.64 to 1.01)	0.02	0.97 (0.94 to 1.00)
Milk chocolate							
**NHS**	Multivariable adjusted	1	0.94 (0.82 to 1.08)	1.05 (0.89 to 1.23)	1.02 (0.72 to 1.44)	0.53	1.00 (0.96 to 1.04)
**NHSII**	Multivariable adjusted	1	0.96 (0.87 to 1.05)	0.97 (0.87 to 1.08)	0.90 (0.72 to 1.12)	0.51	0.98 (0.96 to 1.01)

All models were adjusted for age, calendar year, ethnicity (White, African American, Asian, and others), z-standardized neighborhood socioeconomic status score, smoking status (never, former, current (1–14, 15–24, or ≥25 cigarettes/day), or missing), alcohol intake (gram/day: 0, 0.1-4.9, 5.0-14.9, and ≥15.0 or missing), family history of diabetes (yes/no), menopausal status and post-menopausal hormone use (pre-menopause, post-menopause (never, former, or current hormone use), or missing), use of oral contraceptives (yes, no, NHSII only), physical activity (<3, 3.0-8.9, 9.0-17.9, 18.0-26.9, ≥27.0 metabolic equivalent of task hours/week, or missing), baseline body mass index (BMI) (<21.0, 21.0-22.9, 23.0-24.9, 25.0-26.9, 27.0-29.9, 30.0-32.9, 33.0-34.9, ≥35.0 kg/m^2, or missing), multivitamin use (yes/no), baseline hypertension, baseline hypercholesterolemia, total energy intake, alternative health eating index (quintile groups), total chocolate intake prior to baseline (as cumulative averages between 1980-2002 (NHS), 1991-2003 (NHSII)).

Table S6. Sensitivity analysis further adjusting for educational levels in NHS and specialty of professions in HPFS.

		Chocolate subtypes consumption levels					
		Never or < 1 serving/month	1 serving/month to <1 serving/week	1-4 servings/week	≥5 servings/week	P trend‡	per serving/week
Dark chocolate							
**NHS**	Adjusting for degrees	1	0.92 (0.80 to 1.06)	0.99 (0.83 to 1.17)	0.98 (0.68 to 1.42)	0.92	0.99 (0.95 to 1.04)
**HPFS**	Adjusting for profession	1	1.07 (0.88 to 1.30)	0.93 (0.73 to 1.20)	0.48 (0.26 to 0.90)	0.03	0.91 (0.85 to 0.98)
Milk chocolate							
**NHS**	Adjusting for degrees	1	0.94 (0.83 to 1.08)	1.05 (0.89 to 1.23)	1.02 (0.72 to 1.44)	0.53	1.00 (0.96 to 1.04)
**HPFS**	Adjusting for profession	1	1.05 (0.85 to 1.29)	0.95 (0.74 to 1.22)	0.99 (0.61 to 1.61)	0.98	0.99 (0.93 to 1.05)

All models were adjusted for age, calendar year, ethnicity (White, African American, Asian, and others), smoking status (never, former, current (1–14, 15–24, or ≥25 cigarettes/day), or missing), alcohol intake (gram/day: 0, 0.1-4.9, 5.0-14.9, and ≥15.0 or missing), family history of diabetes (yes/no), menopausal status and post-menopausal hormone use (pre-menopause, post-menopause (never, former, or current hormone use), or missing), use of oral contraceptives (yes, no, NHSII only), physical activity (<3, 3.0-8.9, 9.0-17.9, 18.0-26.9, ≥27.0 metabolic equivalent of task hours/week, or missing), baseline body mass index (BMI) (<21.0, 21.0-22.9, 23.0-24.9, 25.0-26.9, 27.0-29.9, 30.0-32.9, 33.0-34.9, ≥35.0 kg/m^2, or missing), multivitamin use (yes/no), baseline hypertension, baseline hypercholesterolemia, total energy intake, alternative health eating index (quintile groups), total chocolate intake prior to baseline (as cumulative averages between 1980-2002 (NHS), 1991-2003 (NHSII)).
In NHS, the model further adjusted for academic/professional degrees earned (Bachelors, Masters, Doctorate, Registered Nurse).
In HPFS, the model further adjusted for specialty of professions (Dentist, hospital pharmacist, optometrist, osteopath, pharmacist, podiatrist, veterinarian).

Table S7. Weight changes over 4-y periods according to 4-year change in total chocolate intake

	Increase				Decrease				
	β	SE	95% CI	p-values	β	SE	95% CI	p-values	p for interaction
Total Chocolate									
Model 1	0.34	0.02	(0.30 to 0.37)	<0.0001	-0.50	0.02	(-0.54 to -0.47)	<0.0001	/
Model 2	0.25	0.02	(0.22 to 0.29)	<0.0001	-0.40	0.02	(-0.43 to -0.36)	<0.0001	/
Baseline BMI Stratified (kg/m^2)									
BMI<25	0.14	0.02	(-0.22 to -0.15)	<0.0001	-0.18	0.02	(-0.22 to -0.15)	<0.0001	<0.0001
25≤BMI<30	0.29	0.04	(0.22 to 0.36)	<0.0001	-0.56	0.04	(-0.63 to -0.48)	<0.0001	
BMI≥30	0.74	0.09	(0.55 to 0.92)	<0.0001	-1.02	0.1	(-1.21 to -0.83)	<0.0001	

Note: Follow-up periods were 1986–2010 for NHS, 1991–2015 for NHSII, and 1986–2018 for HPFS. β values are interpreted as 4-year weight change in kilograms (95% CI) comparing participants increased or decreased total chocolate intake to those who had no change in total chocolate intake over 4-year periods. Data from three cohorts were combined to run this analysis. p-values were obtained from the Wald test. SE: standard errors. CI: confidence intervals.

Multivariable generalized linear regression models (with independent correlation matrix and robust variance) were used. Baseline BMI stratified models were fully adjusted as Model 2, except that baseline BMI was further adjusted in the continuous form.
Model 1: Adjusted for age.
Model 2: Additionally adjusted for ethnicity (White, African American, Asian, and others), family history of diabetes, baseline hypertension, hypercholesterolemia, baseline and change in total caloric intake, change in smoking status (stayed never smoker, stayed former smoker, stayed current smoker, change from former to current smoker, change from never to current smoker, and change from current to former smoker), baseline and change in physical activity (METs-hour/week), change in alcohol consumption (continuous, g/day), postmenopausal hormone use (women only; premenopausal, never, former, current, or missing), oral contraceptive use (NHSII only; yes, no), changes in alternative healthy eating index (AHEI, quintile groups), and data origin (NHS, NHSII, HPFS).

Table S8. Weight changes over 4-y periods according to 4-year change in chocolate intake, stratified by cohorts.

NHS

	**Increase**				**Decrease**			
	**β**	**SE**	**95% CI**	**p-values**	**β**	**SE**	**95% CI**	**p-values**
Total chocolate[1]								
Model 1	0.29	0.03	(0.24 to 0.34)	<0.0001	-0.47	0.03	(-0.52 to -0.41)	<0.0001
Model 2	0.24	0.03	(0.18 to 0.29)	<0.0001	-0.39	0.03	(-0.45 to -0.34)	<0.0001
Dark chocolate[2]								
Model 1	0.12	0.08	(-0.03 to 0.27)	0.11	-0.19	0.08	(-0.34 to -0.05)	0.01
Model 2	0.09	0.08	(-0.06 to 0.24)	0.26	-0.02	0.08	(-0.18 to 0.14)	0.84
Milk chocolate[2]								
Model 1	0.29	0.08	(0.13 to 0.45)	0.0004	-0.41	0.07	(-0.55 to -0.28)	<0.0001
Model 2	0.26	0.08	(0.10 to 0.43)	0.001	-0.32	0.07	(-0.46 to -0.18)	<0.0001

NHSII

	**Increase**				**Decrease**			
	**β**	**SE**	**95% CI**	**p-values**	**β**	**SE**	**95% CI**	**p-values**
Total chocolate[1]								
Model 1	0.43	0.03	(0.37 to 0.49)	<0.0001	-0.56	0.03	(-0.62 to -0.49)	<0.0001
Model 2	0.33	0.03	(0.27 to 0.39)	<0.0001	-0.41	0.03	(-0.48 to -0.35)	<0.0001
Dark chocolate[2]								
Model 1	0.0003	0.05	(-0.10 to 0.10)	0.996	-0.01	0.04	(-0.10 to 0.08)	0.83
Model 2	-0.09	0.05	(-0.20 to 0.01)	0.08	-0.06	0.06	(-0.17 to 0.05)	0.28
Milk chocolate[2]								
Model 1	0.63	0.06	(0.52 to 0.74)	<0.0001	-0.23	0.04	(-0.32 to -0.15)	<0.0001
Model 2	0.49	0.06	(0.37 to 0.60)	<0.0001	-0.40	0.05	(-0.50 to -0.30)	<0.0001

HPFS

	**Increase**				**Decrease**			
	**β**	**SE**	**95% CI**	**p-values**	**β**	**SE**	**95% CI**	**p-values**

Total chocolate[1]								
Model 1	0.11	0.04	(0.04 to 0.18)	0.002	-0.38	0.03	(-0.45 to -0.32)	<0.0001
Model 2	0.06	0.04	(-0.01 to 0.13)	0.09	-0.34	0.03	(-0.41 to -0.28)	<0.0001
Dark chocolate[2]								
Model 1	-0.13	0.07	(-0.27 to 0.01)	0.07	-0.28	0.08	(-0.44 to -0.12)	0.001
Model 2	-0.12	0.08	(-0.27 to 0.03)	0.11	-0.26	0.08	(-0.42 to -0.09)	0.002
Milk chocolate[2]								
Model 1	-0.02	0.08	(-0.18 to 0.14)	0.83	-0.46	0.08	(-0.61 to -0.32)	<0.0001
Model 2	-0.03	0.08	(-0.19 to 0.14)	0.76	-0.43	0.08	(-0.58 to -0.28)	<0.0001

Note: β values are interpreted as 4-year weight change in kilograms (95% CI) comparing participants increased or decreased total chocolate intake to those who had no change in total chocolate intake over 4-year periods. Data from three cohorts were combined to run this analysis. p-values were obtained from the Wald test. SE: standard errors. CI: confidence intervals.
[1]For total chocolate, follow-up periods were 1986-2010 for NHS, 1991-2015 for NHSII, and 1986-2018 for HPFS.
[2]For dark and milk chocolate, follow-up periods were 2006-2010 for NHS, 2007-2015 for NHSII, and 2006-2018 for HPFS.

Multivariable generalized linear regression models (with independent correlation matrix and robust variance) were used.
Model 1: Adjusted for age.
Model 2: Additionally adjusted for ethnicity (White, African American, Asian, and others), family history of diabetes, baseline hypertension, hypercholesterolemia, baseline and change in total caloric intake, change in smoking status (stayed never smoker, stayed former smoker, stayed current smoker, change from former to current smoker, change from never to current smoker, and change from current to former smoker), baseline and change in physical activity (METs-hour/week), change in alcohol consumption (continuous, g/day), postmenopausal hormone use (women only; premenopausal, never, former, current, or missing), oral contraceptive use (NHSII only; yes, no), and changes in alternative healthy eating index (AHEI, quintile groups).

Figure S1. Participant Flow chart

(a) Total chocolate analysis

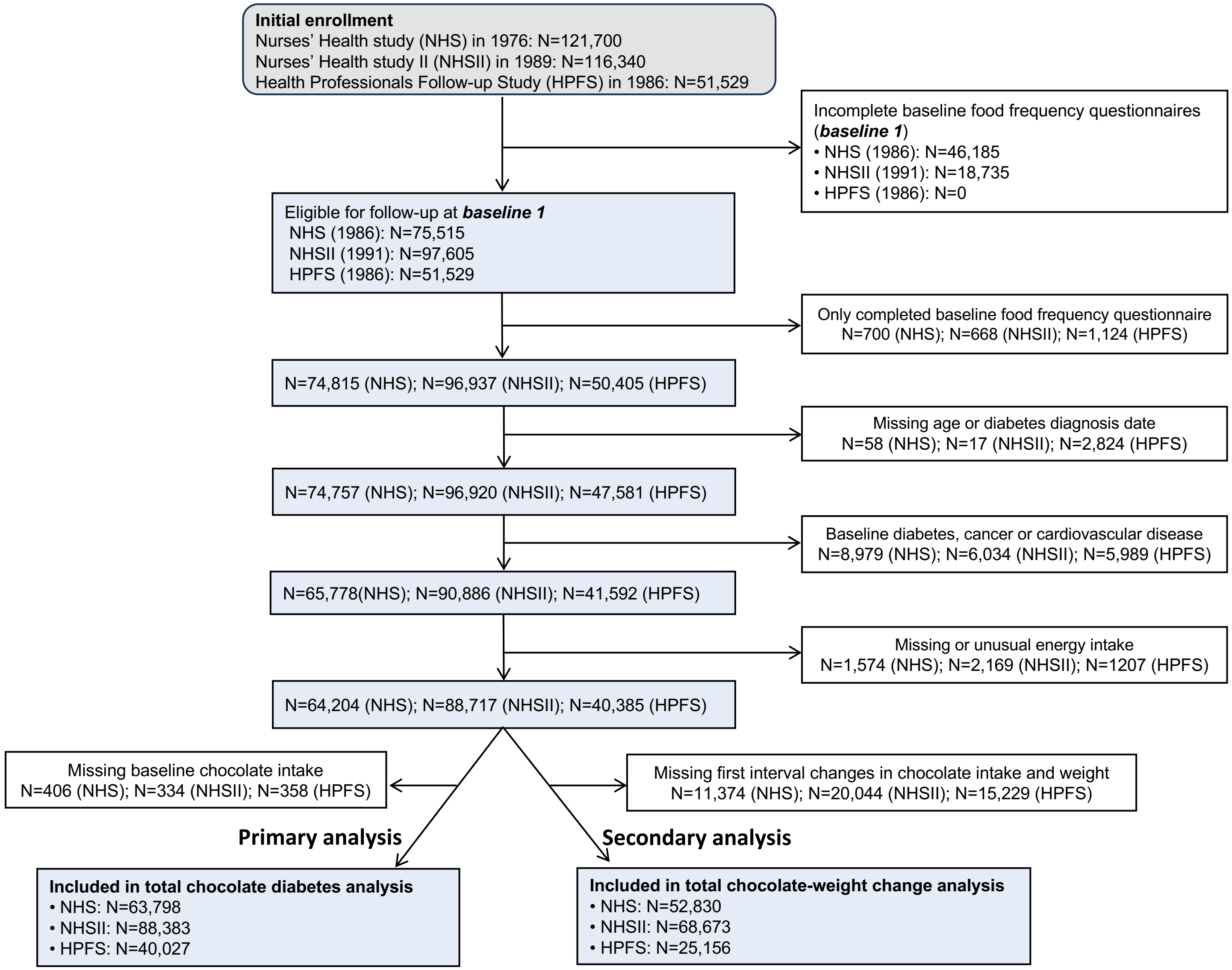

(b) Chocolate subtype analysis

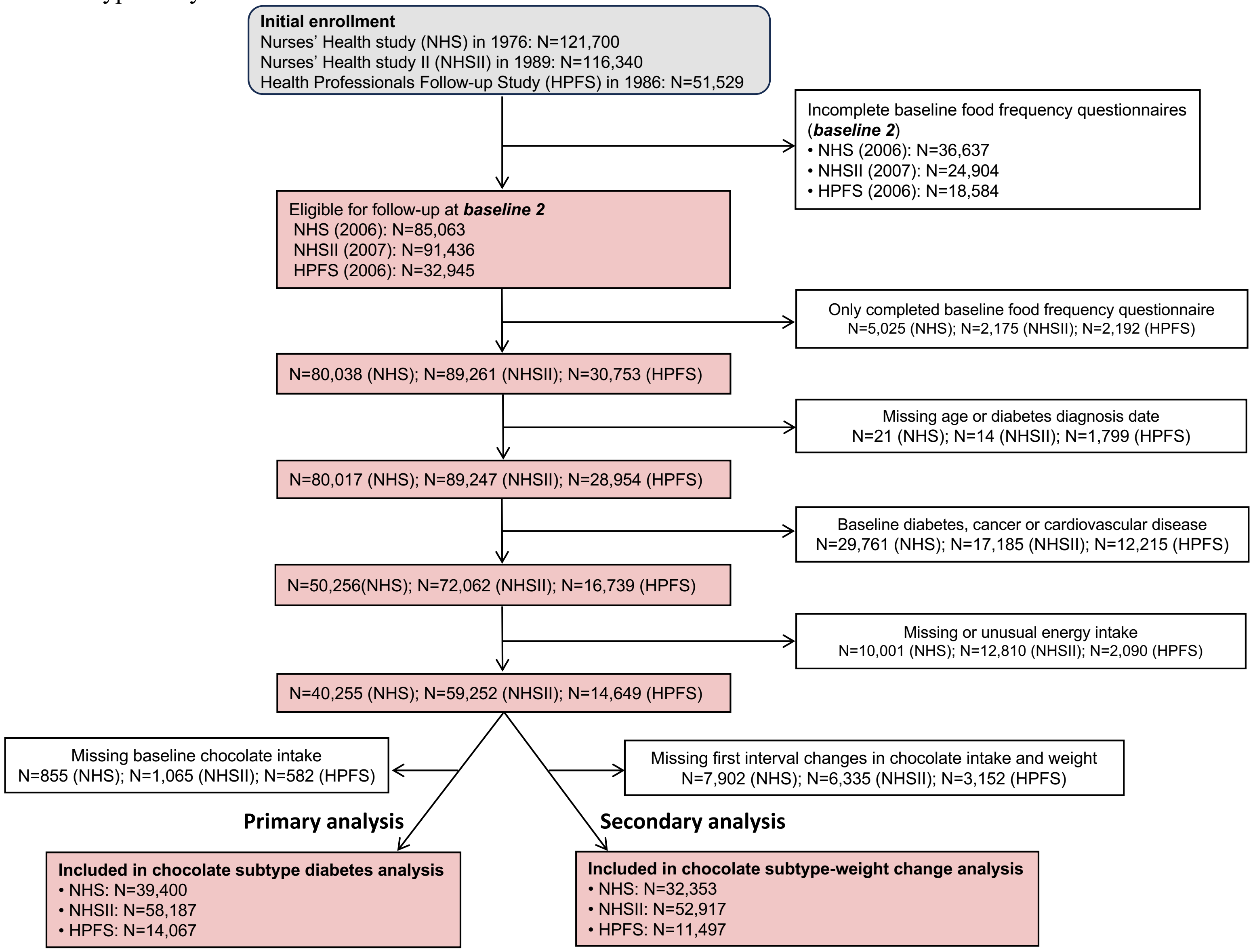

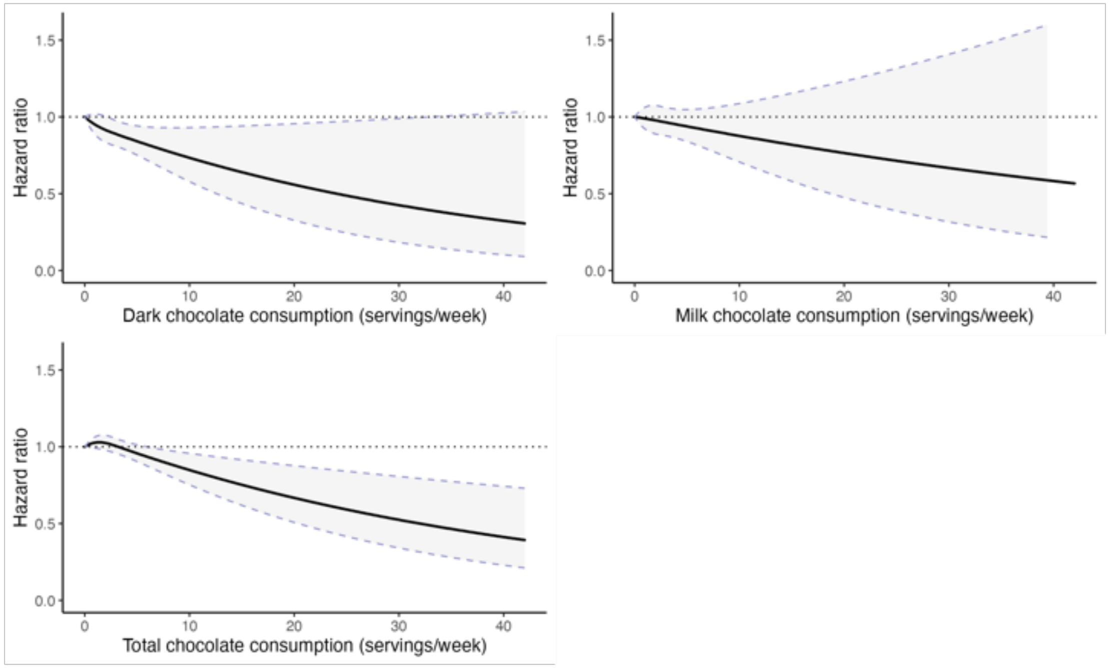

Figure S2. Multivariable adjusted, pooled, dose-response associations between chocolate intake and risk of type 2 diabetes in the Nurses' Health Study (NHS), Nurses' Health Study II (NHSII), and Health Professionals Follow-up Study (HPFS), *not* excluding participants with extreme intake.

Note: Dose-response analysis was performed using SAS Macro %LGTPHCURV9 (number of knots=3). Data from three cohorts were combined.
For dark chocolate, $P_{linearity}$= 0.002, $P_{non\text{-}linearity}$=0.58. For milk chocolate, $P_{linearity}$ = 0.21, $P_{non\text{-}linearity}$ =0.95. For total chocolate, $P_{linearity}$ = 0.03, $P_{non\text{-}linearity}$ =0.02. P values for non-linearity ($P_{non\text{-}linearity}$) were obtained as the significance of the spline terms, and p values for linearity ($P_{linearity}$) were obtained as the significant of the linear terms. P<0.05 indicates statistical significancy.
For dark and milk chocolate, follow-up periods were 2006-2018 for NHS, 2007-2021 for NHSII, 2006-2020 for HPFS. For total chocolate, follow-up periods were 1986-2018 for NHS, 1991-2021 for NHSII, 1986-2020 for HPFS.
Hazard ratios were adjusted for age, calendar year, ethnicity (White, African American, Asian, and others), smoking status (never, former, current (1–14, 15–24, or ≥25 cigarettes/day), or missing), alcohol intake (gram/day: 0, 0.1-4.9, 5.0-14.9, and ≥15.0 in women, 0, 0.1-4.9, 5.0-14.9, 15.0-29.9, and ≥30.0 in men, or missing), family history of diabetes (yes/no), menopausal status and post-menopausal hormone use (pre-menopause, post-menopause (never, former, or current hormone use), or missing, women only), use of oral contraceptives (yes, no, NHSII only), physical activity (<3, 3.0-8.9, 9.0-17.9, 18.0-26.9, ≥27.0 metabolic equivalent of task hours/week, or missing), baseline body mass index (BMI) (<21.0, 21.0-22.9, 23.0-24.9, 25.0-26.9, 27.0-29.9, 30.0-32.9, 33.0-34.9, ≥35.0 kg/m^2, or missing), multivitamin use (yes/no), baseline hypertension, baseline hypercholesterolemia, total energy intake, alternative health eating index (quintile groups), and study origin (NHS, NHSII, HPFS). For dark and milk chocolate dose-response analysis, total chocolate intake prior to baseline (as cumulative averages between 1980-2002 (NHS), 1991-2003 (NHSII), 1986-2002 (HPFS)) were further adjusted.
